# Exosome-Mediated Activation of the Prostasin-Matriptase Serine Protease Cascade in B Lymphoma Cells

**DOI:** 10.3390/cancers15153848

**Published:** 2023-07-28

**Authors:** Li-Mei Chen, Karl X. Chai

**Affiliations:** Burnett School of Biomedical Sciences, College of Medicine, University of Central Florida, Orlando, FL 32816, USA

**Keywords:** exosomes, prostasin, matriptase, protease activation cascade, gelatinase activity, lymphoma

## Abstract

**Simple Summary:**

Prostasin and matriptase are serine proteases co-expressed on the cell membrane in almost all epithelial cells. They reciprocally activate each other to maintain epithelial integrity. In cancers, matriptase is an oncoprotein with key roles in tumor initiation and progression, whereas prostasin acts in the opposite way. A subgroup of Burkitt lymphoma ectopically over-express matriptase without co-expressing prostasin. Reducing the matriptase expression level via small interfering RNAs in the lymphoma cells reduced tumor growth in vitro and in vivo. We hypothesized that an endowment of prostasin in the lymphoma cells can regulate the expression and function of matriptase. We show that prostasin can be introduced to the cancer cells via exosomes to initiate the prostasin–matriptase protease activation cascade and remove matriptase. The method of assembling this protease cascade in B cells via exosomes could be further exploited in animal models for developing alternative treatments for lymphoma.

**Abstract:**

Prostasin and matriptase are extracellular membrane serine proteases with opposing effects in solid epithelial tumors. Matriptase is an oncoprotein that promotes tumor initiation and progression, and prostasin is a tumor suppressor that reduces tumor invasion and metastasis. Previous studies have shown that a subgroup of Burkitt lymphoma have high levels of ectopic matriptase expression but no prostasin. Reducing the matriptase level via small interfering RNAs in B lymphoma cells impeded tumor xenograft growth in mice. Here, we report a novel approach to matriptase regulation in B cancer cells by prostasin via exosomes to initiate a prostasin–matriptase protease activation cascade. The activation and shedding of matriptase were monitored by measuring its quantity and trypsin-like serine protease activity in conditioned media. Sustained activation of the protease cascade in the cells was achieved by the stable expression of prostasin. The B cancer cells with prostasin expression presented phenotypes consistent with its tumor suppressor role, such as reduced growth and increased apoptosis. Prostasin exosomes could be developed as an agent to initiate the prostasin–matriptase cascade for treating B lymphoma with further studies in animal models.

## 1. Introduction

Many physiological and pathophysiological functions are performed by serine proteases, e.g., blood coagulation and fibrinolysis, food digestion, cell apoptosis, and tumor metastasis [1]. These proteolytic enzymes use the hydroxyl group of an active-site serine residue to carry out a nucleophilic attack on the carbonyl group of the scissile peptide bond in their substrates. The results of the substrate cleavage can range from the activation of growth factors or zymogens to protein turnover and tissue remodeling. Most serine proteases, such as the quintessential pancreatic trypsin, are synthesized as precursors (zymogens) and secreted into extracellular spaces or bodily fluids. The secreted zymogens are activated by various factors and mechanisms for various functions. In the past 30 years, several membrane-bound extracellular serine proteases have been discovered, such as hepsin [2], prostasin [3,4], and matriptase [5,6], with specifically defined functions at this cellular localization. The activation and regulation of these enzymes can and also must occur at the membrane, as well.

Prostasin is entirely extracellular, anchored to the membrane via a glycosylphosphatidylinositol (GPI) moiety [7], whereas matriptase is a type-II transmembrane protein with a long carboxyl terminal portion outside the cell, including its serine protease domain [5,6]. Both prostasin and matriptase have been implicated in functional involvements in the initiation and progression of human cancers, but they seem to act in opposite manners. Prostasin is a tumor suppressor, which reduces cancer cell migration, invasion, and metastasis, and its expression level is usually downregulated in cancers [8,9]. Matriptase, on the other hand, is an oncoprotein, and its expression level is usually upregulated in cancers [10]. 

A co-expression of prostasin and matriptase has been observed in almost all normal epithelial cells, in which the pair reciprocally activate each other and maintain the epithelium integrity in a dynamic state [11,12,13,14,15,16,17]. The matriptase zymogen can be activated by prostasin. Subsequently, the activated matriptase can auto-activate additional matriptase zymogen, resulting in matriptase shedding from the cell surface [18,19,20]. Regulation of the prostasin–matriptase proteolytic activation cascade can be achieved via two Kunitz-type transmembrane serine protease inhibitors, hepatocyte growth factor activator inhibitor-1 and -2 (HAI-1 and HAI-2) [19,21,22,23,24]. The active prostasin is presented on the cell surface, whereas the active matriptase is hardly seen and almost always in a complex with HAI-1 on the cell surface or in the extracellular space [7,25,26]. HAI-1 is considered a bona fide inhibitor for matriptase, as its location is on the plasma membrane, bound to the activated matriptase [23]. HAI-2 normally shows an intracellular localization [27,28], with less chance to control the activity of matriptase on the plasma membrane.

Lymphoma is a cancer of the lymphatic system, presenting in two main types, Hodgkin lymphoma and non-Hodgkin lymphoma (NHL). The latter accounts for about 88% of all lymphoma cases and is one of the most common cancers in the United States. There will be 80,550 estimated new cases and 20,180 estimated deaths due to NHL in the United States in 2023 according to the American Cancer Society cancer facts and figures. Lymphoma is also common in children and teens, accounting for about 12% of all childhood cancers. NHL can be subdivided into more than 60 types, including Burkitt lymphoma, which is one of the fastest growing and a very aggressive tumor in humans. Treatment options for NHL are chemotherapy, radiation, and immunotherapy. For recurrent NHL patients or those refractory to the first-line therapy, there is no standard treatment, and the survival rate is rather low (10–30%), presenting an unmet challenge. 

A subgroup of Burkitt lymphoma ectopically over-express matriptase [29,30] without the co-expression of prostasin (this study) typically observed in a normal epithelium. In addition, the two cognate inhibitors of matriptase, HAI-1 and HAI-2, are lacking or expressed at very low levels. An extensive survey was performed on 945 human cancer cell lines for the expression of matriptase and HAI-1 and HAI-2 [31]. Almost all epithelial cancer cell lines expressed both HAI-1 and HAI-2 (98%, 382 out of 391 cell lines), but only 20% of the hematological cancer cell lines (10 out of 51) expressed both HAI-1 and HAI-2 in the same cell line. In hematological cancer cell lines, the levels of HAI-1 and HAI-2 were relatively low. HAI-2 was more frequently co-expressed with matriptase (in 25 out of 51 cell lines, 49%) in the absence of HAI-1. Further, Burkitt lymphoma B cells expressed the highest levels of the matriptase protein and moderate levels of HAI-2 but almost no HAI-1. 

It was hypothesized that the ectopic over-expression of matriptase in blood cancer cells promotes cancer progression. In two Burkitt lymphoma cell lines, Namalwa and Raji, silencing or downregulation of matriptase expression reduced the tumor cell invasion in vitro, reduced tumor growth, and increased apoptosis of xenografts in vivo in SCID mice [29,30]. 

The ectopic over-expression of matriptase in B cell lymphoma is an anomaly in two senses. First, it is a non-epithelial tissue in which matriptase is not normally expressed. Second, it is expressed rather independently from the familiar network of prostasin and HAIs in epithelial tissue, a feature that most likely underscores its tumor-promoting phenotype in B cell lymphoma. Intuitively, these observations invite the question of what would happen if a prostasin co-expression is introduced in the B cells over-expressing matriptase.

As a GPI-anchored protein, prostasin is known to be released into bodily fluids or in tissue culture media in the exosomes [7,32]. Exosomes are small membrane vesicles (30–150 nm in diameter) produced and released by most eukaryotic cells [33]. They contain specific membrane and cellular proteins and nucleic acids depending on the cell origin. Exosomes are capable of merging with other cells via specific receptor–ligand binding, followed by membrane fusion or endocytosis. 

In recent years, prostasin exosomes in circulation or bodily fluids have been studied as potential diagnostic biomarkers in various diseases or conditions, e.g., in the urine of patients with primary aldosteronism, essential hypertension, or albuminuria [34,35,36,37]; in the blood of patients with severe coronavirus disease-2019 (COVID-19) [38]; and in the saliva of patients with oral squamous cell carcinomas [39]. Importantly, prostasin in the exosomes retains its serine protease activity [40,41]. 

In this study, we explore the utility of prostasin exosomes in matriptase activation in B lymphoma cells. Several B lymphoma cell lines over-expressing matriptase were chosen and were co-cultured with prostasin exosomes. Both the prostasin and matriptase serine proteases have a functional domain located outside the plasma membrane, enabling the interaction between the prostasin in the exosomes and the matriptase in the cells. Upon co-culturing, the matriptase content in the cells and in the conditioned media was determined. The activation of the prostasin–matriptase cascade was monitored by measuring the serine protease activity in the conditioned media. The migration and invasion abilities of the B lymphoma cells were examined during the prostasin–matriptase cascade activation. 

## 2. Materials and Methods

### 2.1. Cell Culture

The human Burkitt lymphoma cell lines Daudi (ATCC^®^ CCL-213™), Namalwa (ATCC^®^ CRL-1432™), Ramos (RA 1) (ATCC^®^ CRL-1596™), Raji (ATCC^®^ CCL-86™), the JeKo-1 (ATCC^®^ CRL-3006™) human mantle cell lymphoma cell line, and the RS4;11 (ATCC^®^ CRL-1432™) human acute lymphoblastic leukemia cell line were purchased from the ATCC (American Type Culture Collection, Manassas, VA, USA). All cells were maintained according to the ATCC instructions in an incubator at 37 °C with a humidified atmosphere of 5% CO_2_ in air. Tissue culture flasks and dishes were purchased from Sarstedt, Inc. (Newton, NC, USA). Heat-inactivated fetal bovine serum (FBS) was purchased from Sigma-Aldrich (St. Louis, MO, USA). Other cell culture media and reagents were purchased from Thermo Fisher Scientific (Waltham, MA, USA). 

### 2.2. Establishment of an HEK293T Subline Over-Expressing Prostasin for Exosome Production

The HEK293T-Pro and HEK293T-Vec sublines expressing human prostasin or carrying empty pLVX-Puro vector (Clontech laboratories, Inc., Mountain View, CA, USA) were produced from the HEK293T (ATCC^®^ CRL-3216™) human embryonic kidney cells using lentiviruses and procedures described previously [42]. The human prostasin protein was confirmed to be located on the cell-surface membrane of nearly 100% of the prostasin-expressing cells, as determined by flow cytometry. 

We also established a tetracycline-regulated prostasin expression in the Namalwa cells (NamalwaTR-Pro, or NamalwaTR-ProM, a serine active-site mutant) using a previously described method [43]. Cells with the vector alone were used as the control (NamalwaTR-Vec). 

### 2.3. Exosome Isolation and Cell Treatment 

The HEK293T subline cells were cultured to confluence, and the conditioned media were collected. The conditioned media were centrifuged to remove cell debris and large vesicles and subjected to the PEG method of exosome isolation, as described in [41]. The exosome pellets were resuspended in phosphate-buffered saline (PBS) and further purified by ultracentrifugation at 100,000× *g* for 90 min to remove soluble protein carryovers from the culture medium. The exosome total protein concentration was determined using the Pierce™ BCA Protein Assay Kit (Thermo Fisher Scientific, Waltham, MA, USA). Calu-3 (ATCC^®^ HTB-55™) human lung adenocarcinoma cells and derivative cells expressing various forms of prostasin, or having the prostasin gene knocked-out, were cultured as described previously [41]. B cancer cells were collected and resuspended in an assay buffer (2%FBS in RPMI medium). The amount of exosomes from 1 mL of the culture medium was defined as 1 unit. For cell–exosome co-cultures, the isolated exosomes were added at a ratio of 1 unit per 1 × 10^6^ cells, with the final exosome concentration by total protein at 25–30 μg/mL. The cell–exosome mixture was incubated at 37 °C overnight. 

### 2.4. Reverse Transcription and Real-Time Quantitative Polymerase Chain Reaction (RT-qPCR)

The procedures were carried out as described previously [41]. One microgram of total RNA from each sample was used for reverse transcription using the iScript reagent kit (Bio-Rad, Hercules, CA, USA), and one-fifth of the iScript product was used for each gene-specific qPCR. For quantitative comparison between samples, the relative expression levels were compared using the glyceraldehyde 3-phosphate dehydrogenase (GAPDH) copy number as the reference.

### 2.5. SDS-Polyacrylamide Gel Electrophoresis (PAGE), Western Blot Analysis, Gelatin-Gel Zymography

SDS-PAGE and western blot analysis were performed as described previously [41]. The original western blot figures could be found in File S1. All samples were mixed with the Laemmli sample buffer, including reducing agents, and boiled before electrophoresis. For western blot analysis, the following antibodies were used: prostasin [7]; matriptase (sc-365482, monoclonal, Santa Cruz Biotechnology, Inc., Dallas, TX, USA, or A300-221A, polyclonal, Bethyl Laboratories, Montgomery, TX, USA, or AF3946, polyclonal, R&D Systems, Inc., Minneapolis, MN, USA); HAI-2 (AF1106, R&D Systems, Inc.); and GAPDH (Santa Cruz Biotechnology). The western blot images were captured using a ChemiDoc MP Imager and analyzed with Image Lab software 6.1 (Bio-Rad).

Gelatin zymography was carried out as described previously [44]. Briefly, gelatin (Sigma) at a final concentration of 0.1% was incorporated into SDS-polyacrylamide gel (7.5–10%). Cell lysate and conditioned media were incubated with non-reducing Laemmli sample buffer at room temperature for 15 min and were resolved on the gelatin gel. After electrophoresis, the samples in the gel were renatured in a 2% Triton^®^ X-100 (Thermo Fisher) solution with agitation for 1 h with one solution change. The gel was then incubated overnight in a buffer of 50 mM Tris at pH 8.0, containing 137 mM NaCl and 5 mM CaCl_2_. The gel was stained with 0.25% Coomassie blue for 30–60 min, destained, and imaged using the ChemiDoc MP Imager.

### 2.6. Protease Activity Assay

Trypsin-like serine protease activity was measured using a synthetic tripeptide substrate Gln-Ala-Arg (QAR) with the fluorogenic leaving group AMC (Boc-QAR-AMC, R&D Systems). The conditioned media were collected by centrifugation at 24 h of co-culturing. In a 96-well plate, 10 μL of each medium sample were mixed with 90 µL of 0.1 M Tris at pH 8.5, containing 100 mM NaCl and 20 µM of the QAR substrate. The mixtures were read immediately using a SpectraMax i3x Multi-Mode Microplate Reader (Molecular Devices, San Jose, CA, USA) in the kinetic mode at 380 nm/480 nm excitation/emission for 2 h, with intervals of 2 min between readings.

### 2.7. Flow Cytometry

Flow cytometry was performed as described previously [41] with some modifications. A human Fc receptor-binding inhibitor polyclonal antibody (50-112-9053, Fisher Scientific) was used as a blocking agent prior to adding the primary antibodies in all B cell surface-labeling experiments. Rabbit anti-human prostasin sera or pre-immune rabbit sera [7] and the matriptase antibody (A6135, ABclonal Technology, Woburn, MA, USA) were used as the primary antibodies at 1:100 dilution. A goat anti-rabbit IgG-cyanine-Cy™3 (Jackson ImmunoResearch Laboratories, Inc., West Grove, PA, USA) was used as the secondary antibody at 1:200 dilution. The labeled cells (10,000) were analyzed using a CytoFLEX S flow cytometer with the laser configuration of V2B2Y3R2, operated by CytExpert software v2.3 (Beckman Coulter, Brea, CA, USA). The data were analyzed with FlowJo™ software v10.8.1.

### 2.8. Migration and Invasion

Transwell^®^ inserts for migration and invasion were purchased from Corning Inc. (Corning, NY, USA). Human chemokines SDF-1 and CXCL 13 (BCA-1) were purchased from ProSpec (Rehovot, Israel). Chemoattractant-induced cell migration assays were performed according to procedures described previously [45] with modifications, using 5 µm Transwell cartridges (Corning, cat. No. 3421). Cells were washed with RPMI medium and incubated with exosomes at a ratio of 1 unit per 1 million cells in 100 μL of RPMI medium. The cell–exosome mixture was incubated for 2–3 h at 37 °C before seeding into the Transwells (3 × 10^5^/100 µL per Transwell). The bottom well contained the growth medium with BCA-1 and SDF-1, at 10 ng/mL and 50 ng/mL, respectively. After 24 h of incubation, the cells in the bottom chamber were counted using the CytoFLEX S flow cytometer. The data were analyzed with FlowJo software v10.8.1. The invasion assay was performed the same way as the migration assay, with 50 µL of Matrigel^®^ Matrix (Corning, Corning, NY, USA) added in the Transwell cartridge and gelled at 37 °C for 2 h before seeding the cells on top of the gelled matrix.

### 2.9. Statistical Analysis

Data were analyzed in GraphPad Prism 9 or Excel and were expressed as mean ± standard errors (SE). A student’s *t* test was used to compare the means between two groups, in which a *p* value less than 0.05 was considered statistically significant. One-way analysis of variance (ANOVA) coupled with the Tukey post hoc test was used to determine statistical significance when comparing three or more independent groups, in which a *p* value less than 0.05 was considered statistically significant. 

## 3. Results

### 3.1. Expression of Matriptase and Hepatocyte Growth Factor Activator Inhibitors (HAIs) in B Lymphoma Cell Lines

**Figure 1:**
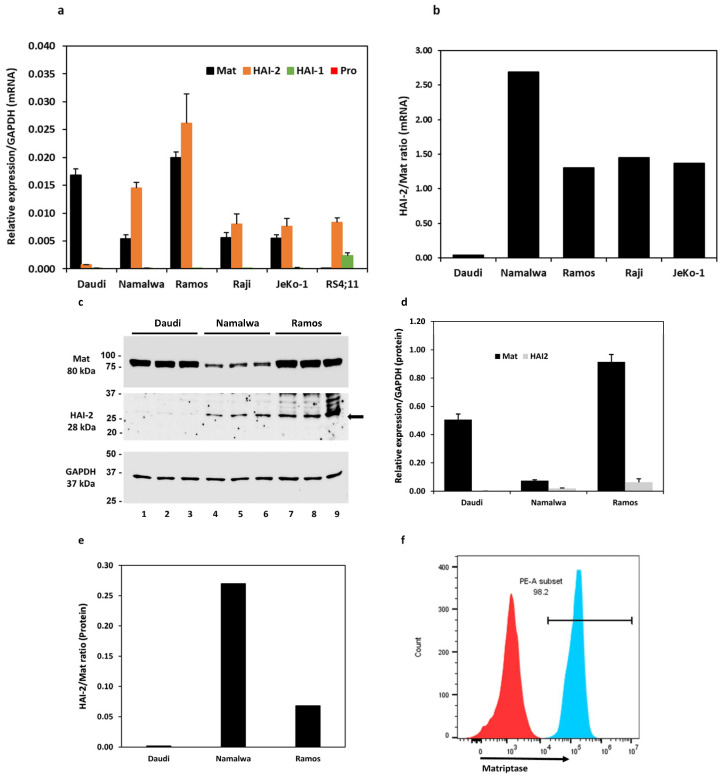
Expression analysis of matriptase, HAI-1, HAI-2, and prostasin in B cancer cells by reverse-transcription/qPCR (**a**,**b**), western blotting (**c**–**e**), and flow cytometry (**f**). (**a**) Bar graph of relative mRNA expression levels of matriptase (Mat), HAI-1, HAI-2, prostasin (Pro) in Daudi (*n* = 4), Namalwa (*n* = 5), Ramos (*n* = 3), Raji (*n* = 3), JeKo-1 (*n* = 3), and RS4;11 cells (*n* = 2) using GAPDH as the reference. The prostasin bars do not appear in the bar graph, as the actual qPCR readouts were registered as “N/A” by the instrument. (**b**) Bar graph of mRNA quantity ratio of HAI-2 to matriptase after normalization with the GAPDH level in each cell line in (**a**). (**c**) Western blotting images of matriptase (Ab: A300-221A), HAI-2, and GAPDH. Twenty micrograms of total protein from the cell lysate of each individual culture (including 2 repeats) were analyzed. Daudi, lanes 1–3; Namalwa, lanes 4–6; Ramos, lanes 7–9. Top panel, matriptase (Mat); middle panel, HAI-2; bottom panel, GAPDH. (**d**) Densitometry bar graph of relative protein quantities of matriptase and HAI-2 using GAPDH as the reference. (**e**) The quantitative ratio of HAI-2 to matriptase in each cell line. (**f**) Flow cytometry histogram of matriptase expression evaluation in Ramos cells. The Ramos cells (4 × 10^5^) were labeled with the matriptase antibody as described in the Materials and Methods section. The matriptase-positive cells are shown in the PE-A subset (blue peak). Cells without the matriptase antibody labeling (red peak) were not detected in the PE-A subset and were used as the gating control.

Six cell lines, including four Burkitt lymphoma cell lines, Daudi, Namalwa, Ramos, Raji, a mantle cell lymphoma cell line JeKo-1, and an acute lymphoblastic leukemia cell line RS4;11 (referred to as RS4 hereon), were evaluated for the expression of matriptase, prostasin, and HAIs at the mRNA level. As shown in Figure 1a, all four Burkitt lymphoma cell lines and the JeKo-1 cells express the matriptase (Mat) and HAI-2 mRNAs, but not the HAI-1 or the prostasin mRNA. The RS4 cells do not express matriptase or prostasin but have detectable amounts of HAI-1 and HAI-2 mRNAs. The quantitative ratio of HAI-2 to matriptase (HAI-2/Mat) differed over a wide range (Figure 1b): 0.04 in the Daudi, 2.69 in the Namalwa, 1.31 in the Ramos, 1.45 in the Raji, and 1.37 in the JeKo-1 cells. 

The matriptase protein levels were analyzed in the Daudi, Namalwa, and Ramos cells, and the ratios of HAI-2 to matriptase were 0.002 in the Daudi, 0.27 in the Namalwa, and 0.07 in the Ramos cells (Figure 1c–e). The matriptase protein expression pattern is similar to that at the mRNA level. The matriptase protein levels are relatively high in the Daudi and the Ramos but low in the Namalwa cells. The HAI-2 level is high in the Ramos but hardly detectable in the Daudi cells. The proteolytic activity of matriptase may be better controlled by HAI-2 in the Namalwa cells than in the Ramos cells, but it is not well-controlled in the Daudi cells. These results are in agreement with previous studies [30,31]. 

We further showed by flow cytometry that the matriptase protein is localized on the cell surface of Ramos cells. The Ramos cells were fixed without permeabilization and labeled with a polyclonal matriptase antibody, followed by the secondary antibody conjugated with the fluorophore Cy3 (Figure 1f, PE-A subset/blue peak). Matriptase was localized on the cell surface in 98.2% of the live singlet Ramos cells. The cells that went through the labeling procedures without the matriptase antibody were used as the gating control (Figure 1f, red peak). The matriptase protein in these B lymphoma cells is transported to the cell surface and has an opportunity to interact with its substrates, e.g., prostasin, or inhibitors, e.g., the HAIs.

### 3.2. Prostasin Exosomes Reduce the Matriptase Protein Level in B Lymphoma Cells

**Figure 2:**
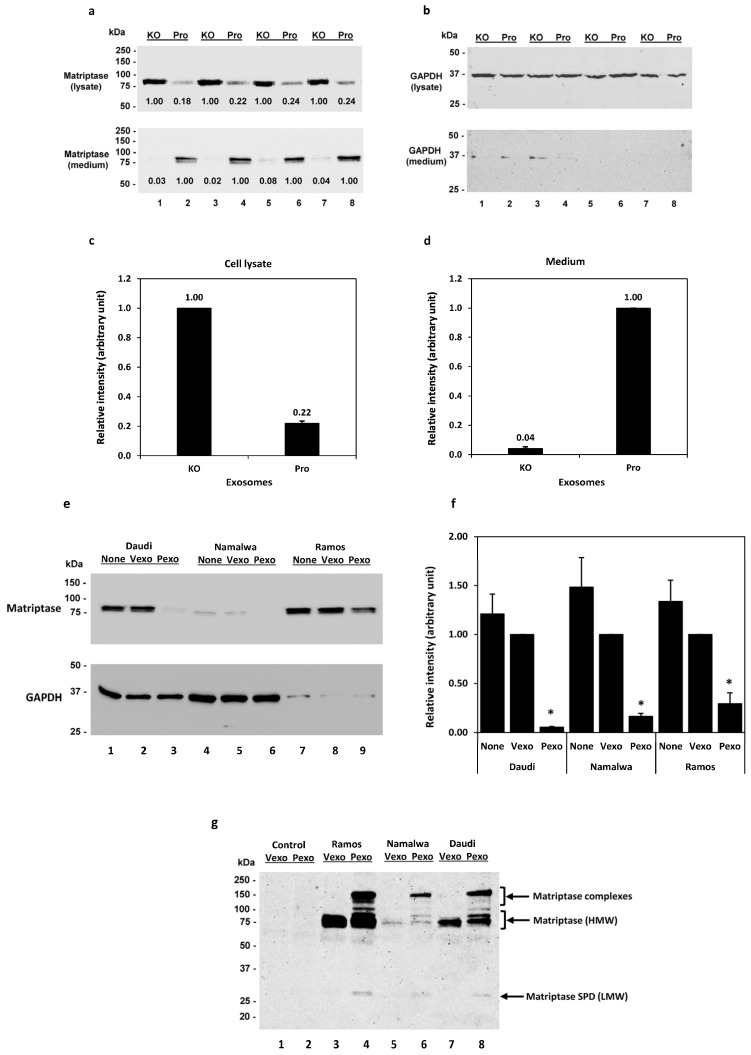
Prostasin exosomes reduce matriptase quantity in B cancer cells. (**a**) Western blot images of matriptase (Ab: A300-221A) in samples from the cell lysate (top panel) and the conditioned media (bottom panel) after incubation with prostasin exosomes (Pro) or exosomes without prostasin (KO). The Daudi cells (2 × 10^5^ cells each) were incubated with the exosomes in 50 μL of OPTI-MEM I/2%FBS (lanes 1–4) or RPMI medium (lanes 5–8) overnight. One-half of each cell lysate or 40 μL of each media supernatant were analyzed. (**b**) Western blot images of GAPDH from (**a**). (**c**) Densitometry of relative intensities of matriptase in the cell lysate or media (**d**). Data presented are the average intensity of lanes 1, 3, 5, 7 versus that of lanes 2, 4, 6, 8 after normalization with GAPDH in (**b**). (**e**) Western blot images of matriptase (top panel; Ab: sc-365482) in the Daudi, Namalwa, and Ramos cells treated with exosomes isolated from the HEK293T cells. Cells (2.5 × 10^5^) were co-cultured with prostasin exosomes (Pexo, lanes 3, 6, 9) or vector exosomes (Vexo, lanes 2, 5, 8) in 100 μL of OPTI-MEM I/2%FBS. Cells without exosomes (None, lanes 1, 4, 7) were cultured in the same conditions. Bottom, GAPDH western blot image. (**f**) Bar graph of (**e**) expressed as the relative intensities of matriptase in cells treated with vector exosomes (Vexo), prostasin exosomes (Pexo), or no exosomes (None) using GAPDH as the reference. * denotes *p* < 0.05 between Vexo and Pexo. (**g**) Western blot image of matriptase (Ab: AF3946) in conditioned media (20 μL each) of samples in (**e**) treated with exosomes. The AF3946 human matriptase/ST14 catalytic domain antibody recognized a 28–30 kDa band in the Pexo-treated sample media. This antibody also recognized the high-molecular-weight (HMW) matriptase at 80-kDa as well as unknown/uncharacterized matriptase complexes in the Pexo-treated sample media. The Vexo and Pexo samples alone were included in the blot as controls, in which no specific proteins were recognized by this antibody.

We have previously shown in Calu-3 human lung adenocarcinoma cells that the prostasin protein can be released into the culturing medium, retaining its serine protease activity in the lipid bilayer of exosomes [41]. Prostasin has been reported to activate matriptase [40,42,46,47,48]. Here, we show that adding prostasin exosomes in the Daudi cell culture activated matriptase and released matriptase into the culturing medium (Figure 2). Prostasin-enriched exosomes were isolated from the conditioned medium of Calu-3 cells over-expressing prostasin (Pro), whereas prostasin-depleted exosomes were isolated from Calu-3 cells lacking prostasin expression as a result of CRISPR/Cas9 mediated gene knockout (KO) [41]. As shown in Figure 2a (top panel), upon incubation with the prostasin exosomes (Pro, lanes 2, 4, 6, 8), the quantity of matriptase in the Daudi cells was greatly reduced to about 22% of that in the cells incubated with the exosomes without prostasin (KO, lanes 1, 3, 5, 7). Conversely, the amount of matriptase released into the medium of cells incubated with the prostasin KO exosomes was only about 4% of that in the medium of cells incubated with the prostasin exosomes. The amounts of matriptase in the cell lysate and media are inversely correlated (Figure 2c,d). This result indicated that prostasin can devolve matriptase from the cell surface into the medium. The GAPDH protein was detected in all cell lysate samples but barely detectable in the media (Figure 2b). 

Similarly, as shown in Figure 2e, the prostasin-enriched exosomes (Pexo) isolated from the HEK293T cells over-expressing prostasin greatly reduced the matriptase amount in the Daudi (lane 3), the Namalwa (lane 6), and the Ramos (lane 9) cells, in comparison to the cells treated with the vector control exosomes (Vexo) isolated from the HEK293T cells harboring the empty lentiviral vector (lanes 2, 5, 8). Cells treated with the Vexo exosomes appeared to have a lower matriptase level than cells without the exosome treatment (None, lanes 1, 4, 7), but the difference was not statically significant (*p* > 0.05). The HEK293T exosomes were validated using exosome markers, and the exosomal prostasin was shown to be active using an established protease nexin-1 binding assay [7] (Figure S1).

These results suggest that matriptase ectopically expressed in B cancer cells was activated by the prostasin exosomes and shed from the cells, similar to observations described for epithelial cells [18,19,20]. The event of matriptase activation and shedding from the cells is accompanied by the release of a low-molecular-weight (LMW) fragment (28–30 kDa) in the medium. As shown in Figure 2g, this LMW matriptase fragment was detected more prominently in the conditioned media of cells treated with the prostasin exosomes (Pexo, lanes 4, 6, 8), in comparison to that of cells treated with the vector control exosomes (Vexo, lanes 3, 5, 7). The matriptase profile, including the matriptase complexes, in the conditioned media is very similar to that reported previously for these B lymphoma cell lines upon acid activation of matriptase [31].

### 3.3. The GPI Anchor and the Serine Active Site of Prostasin Play Roles in the Reduction of Matriptase Quantity in B Lymphoma Cells

**Figure 3:**
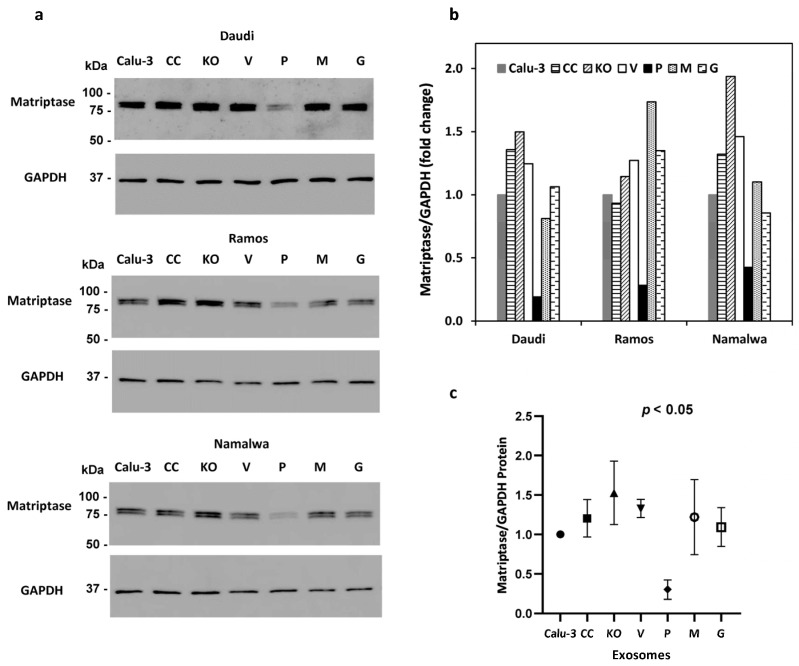
B cell matriptase quantity reduction by wild-type prostasin. (**a**) Western blot images of matriptase (Ab: sc-365482) and GAPDH in the Daudi (top two panels), Ramos (middle two panels), and Namalwa (bottom two panels) cells treated with exosomes isolated from the Calu-3 cells and sublines with over-expressed prostasin or variants. Calu-3, parent cells; KO, subline with prostasin gene knockout; P, subline with the wild-type prostasin; M, subline with a serine active-site mutant prostasin; G, subline with an active prostasin without the GPI anchor; CC and V are control sublines for KO and P,M,G, respectively. The intensity of each band was quantified using Image Lab 6.1 software (Bio-Rad), normalized against the corresponding GAPDH signal, and is shown in the bar graph (**b**). (**c**) Effects of various prostasin exosomes on matriptase quantity reduction in B cancer cells. Data from (**b**) were combined and analyzed in GraphPad Prism 9 and are shown in the dot plot. ANOVA *p* < 0.05.

Exosomes carrying over-expressed prostasin variants were isolated from a series of Calu-3 sublines as previously described [41] and incubated with the Daudi, Ramos, and Namalwa cells. The amount of cell-associated matriptase was analyzed by SDS-PAGE/western blotting and quantified by densitometry using GAPDH as the reference. The amount of matriptase was much less in the Daudi, Ramos, and Namalwa cells treated with exosomes carrying the GPI-anchored active prostasin (P) than in the cells treated with exosomes carrying a prostasin active-site mutant (M) or exosomes from cells expressing a GPI-anchor-free prostasin (G). The GPI-anchor-free prostasin is secreted and not expected to be present on the exosomes. Exosomes isolated from other Calu-3 sublines, including the prostasin-knockout (KO) and its control (CC), and the control subline (V) for the over-expression lines (P, M, G) did not significantly reduce the matriptase level in the B lymphoma cells (Figure 3a,b). The wild-type prostasin with both the membrane anchorage and the serine active site provided the most robust matriptase removal power (Figure 3c).

### 3.4. Trypsin-like Serine Protease Activity Is Increased in the Conditioned Media of Cells Treated with Prostasin Exosomes

**Figure 4:**
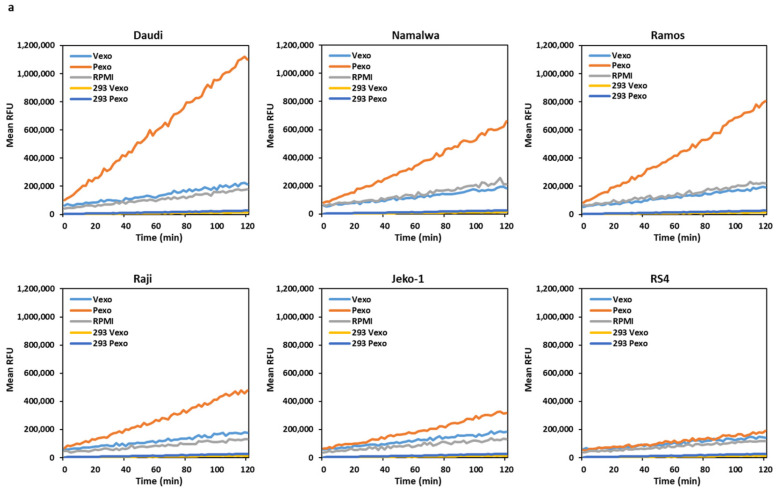

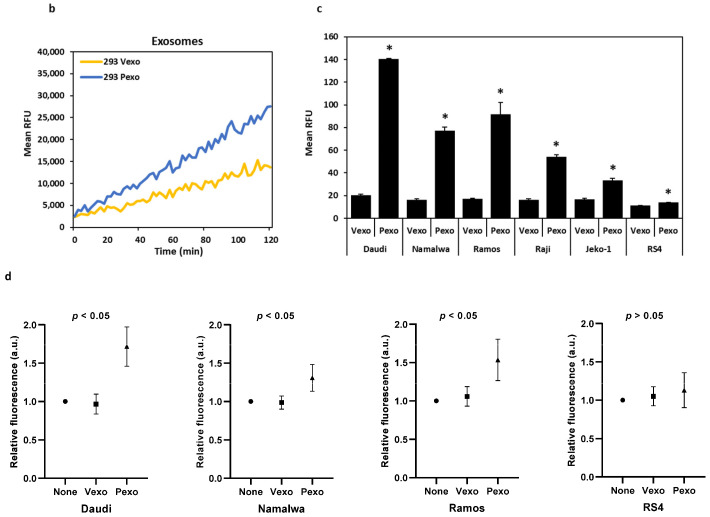
Trypsin-like serine protease activity measurement. (**a**) Line graphs of trypsin-like serine protease activity expressed as relative fluorescent units (RFU). After mixing 2 × 10^6^ cells with exosomes (Vexo or Pexo) or with RPMI medium alone in a total volume of 500 μL for 24 h, the activity in the conditioned medium was measured continually for 120 min. (**b**) Activity graph of Vexo or Pexo exosomes from the HEK293T-Vec or Pro cells in RPMI medium without cells, used as background controls. (**c**) Bar graph of data from (**a**) after subtracting the background controls of (**b**). * denotes *p* < 0.05. (**d**) Dot plots of trypsin-like serine protease activity in media collected 10 days after cell–exosome co-culturing. a.u., arbitrary units. Data were analyzed in GraphPad Prism 9. ANOVA, *p* < 0.05 for Daudi, Namalwa, and Ramos, *p* > 0.05 for RS4.

The Daudi, Namalwa, Ramos, Raji, Jeko, and RS4 cells were co-cultured overnight with prostasin exosomes (Pexo) or the vector control exosomes (Vexo) prepared from the HEK293T-Pro or Vec cells, respectively. Cells without any addition of exosomes were also cultured in the same conditions. As shown in Figure 4a,c, the conditioned media from the cells treated with the prostasin exosomes (Pexo) had a higher trypsin-like serine protease activity than that of cells treated with the vector exosomes (Vexo). The highest activity was detected in the Pexo media of the Daudi cells, coinciding with the high matriptase expression level in these cells (Figure 1a). The lowest activity was in the Pexo media of the Jeko-1 cells, coinciding with a lower matriptase expression level (Figure 1a). The media of cells with the Vexo or no exosome (RPMI) have similar and low levels of protease activity (Figure 4a). The Vexo or Pexo sample with the exosomes alone also has measurable serine protease activity (Figure 4b) but at much lower levels. The activity of Pexo measures at ~2.3% of that in the Daudi conditioned media or ~9% of that in the Jeko-1 conditioned media from co-culturing with Pexo. This low level of protease activity is attributed to the intrinsic serine protease activity of the exosomes, especially in Pexo [7,41].

In duplicate experiments, we showed that upon prostasin exosome treatment, the elevated trypsin-like serine protease activity remained high for at least 10 days (Figure 4d). This phenomenon was only seen for the matriptase-expressing cells, but not the RS4 cells, which do not express matriptase. These results suggest that the increased trypsin-like serine protease activity may be attributed to the increased amount of matriptase released into the conditioned media after the prostasin exosome treatment.

### 3.5. Over-Expression of Prostasin Reduces Matriptase Quantity in B Cells

**Figure 5:**
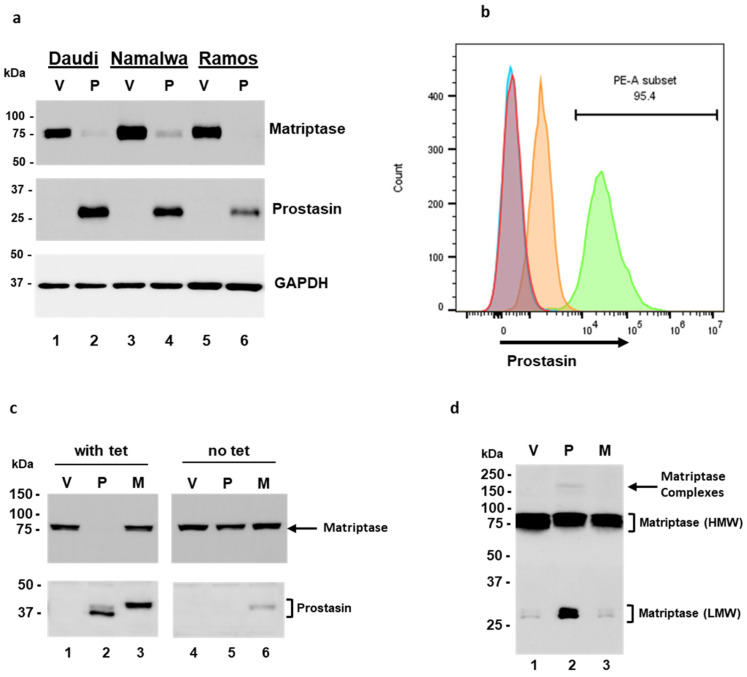
Ectopic expression of prostasin in B cancer cells. (**a**) Western blot analysis of transient expression of prostasin (P) or vector alone (V) in the Daudi, Namalwa, and Ramos cells. The lysate from 2 × 10^5^ cells of each type was analyzed. Top panel, matriptase (Ab: sc-365482); middle panel, prostasin; bottom panel, GAPDH. (**b**) Flow cytometry analysis of Namalwa sublines with tetracycline-induced prostasin expression or vector alone. Red peak (vector-alone cells) and sky-blue peak (prostasin-expressing cells) are samples without the prostasin antibody incubation. Orange peak (vector-alone cells) and green peak (prostasin-expressing cells) are samples incubated with the prostasin antibody. All samples were incubated with a secondary antibody conjugated with the fluorophore Cy3, and 10,000 cells of each sample were analyzed in a CytoFLEX S flow cytometer. The data were analyzed with FlowJo™ software v10.8.1 and are presented in the histogram. (**c**) Western blot analysis of NamalwaTR sublines. One hundred thousand cells of each sample were analyzed. Lanes 1 and 4 or V, samples of the vector control subline; lanes 2 and 5 or P, samples of the subline with the wild-type prostasin; lanes 3 and 6 or M, samples of the subline with a serine active-site mutant prostasin. Left panel, cells were grown in OPTI-MEM I/2%FBS with 1 μg/mL tetracycline (with tet); right panel, cells were grown without tetracycline (no tet) for 8 days. Top two panels, matriptase antibody (sc-365482); bottom two panels, prostasin antibody. (**d**) Western blot analysis of tet-conditioned media from (**c**). Two hundred milliliters of the conditioned media were precipitated with trichloroacetic acid (TCA) (final 16.7%) at 4 °C overnight. The pellet was collected via centrifugation and analyzed. The membrane was blotted with the AF3946 human matriptase/ST14 catalytic domain antibody.

The Daudi, Namalwa, and Ramos cells were transduced with a lentivirus harboring the human prostasin cDNA for over-expression [42]. The cells were collected 24 h post-infection, lysed, and subjected to SDS-PAGE/western blot analysis using antibodies against matriptase, prostasin, or GAPDH, respectively. In cells with prostasin over-expression (Figure 5a, middle panel, lanes 2, 4, 6), the matriptase expression was almost abolished, in comparison to the cells without prostasin (Figure 5a, top panel, lanes 1, 3, 5). The GAPDH contents were used as the loading controls (Figure 5a, bottom panel). 

In the NamalwaTR sublines, upon tetracycline induction, the NamalwaTR-Pro cells expressed a great amount of prostasin at the cell surface with the GPI anchor (Figure 5b, green peak), whereas the NamalwaTR-Vec cells did not express prostasin (Figure 5b, orange peak). At least 95.4% of the NamalwaTR cells were shown by flow cytometry to express prostasin after the tetracycline induction, as indicated by the right-shifted peak (PE-A subset/green peak), representing the cells labeled with a polyclonal prostasin antibody and the fluorophore-conjugated secondary antibody. The tetracycline-treated NamalwaTR-Vec (red peak) and NamalwaTR-Pro (sky-blue peak) cells without the prostasin antibody incubation that went through the same labeling procedures were negative for prostasin staining. Their representative peaks appear to the far left on the x-axis. The ectopically expressed prostasin protein in the B cells was correctly transported on the B cell surface.

In the long-term culture of tet-regulated NamalwaTR sublines, a sustained presence of the wild-type prostasin abolished the cellular matriptase presence (Figure 5c, lane 2), but the serine active-site mutant prostasin did not elicit such an effect (Figure 5c, lane 3). The cells cultured without tetracycline were analyzed and run as controls, wherein the cellular matriptase level appeared similar across the sublines (Figure 5c, lanes 4, 5, 6). The wild-type prostasin expressed in the NamalwaTR-Pro cells appeared in two bands, indicating a sustained activation. The conditioned media of these sublines in the long-term tet-induced culture also reproduced the shed-off matriptase profile (Figure 5d). The NamalwaTR-Pro cell medium had a much higher quantity of LMW matriptase, in comparison to that of the NamalwaTR-Vec or ProM cells.

### 3.6. Proliferation, Migration, Invasion, and Gelatinase Activity Changes Associated with Prostasin

We have shown in Figure 2, Figure 3 and Figure 5 that prostasin reduced the matriptase level in B cancer cells, similar to the observations in epithelial cells in which prostasin over-expression reduced cellular matriptase levels [42,47]. We set out experiments to determine whether the prostasin-exosome-mediated reduction in the matriptase level impacts the growth, migration, and invasion properties of the B cancer cells.

#### 3.6.1. Proliferation and Apoptosis

**Figure 6:**
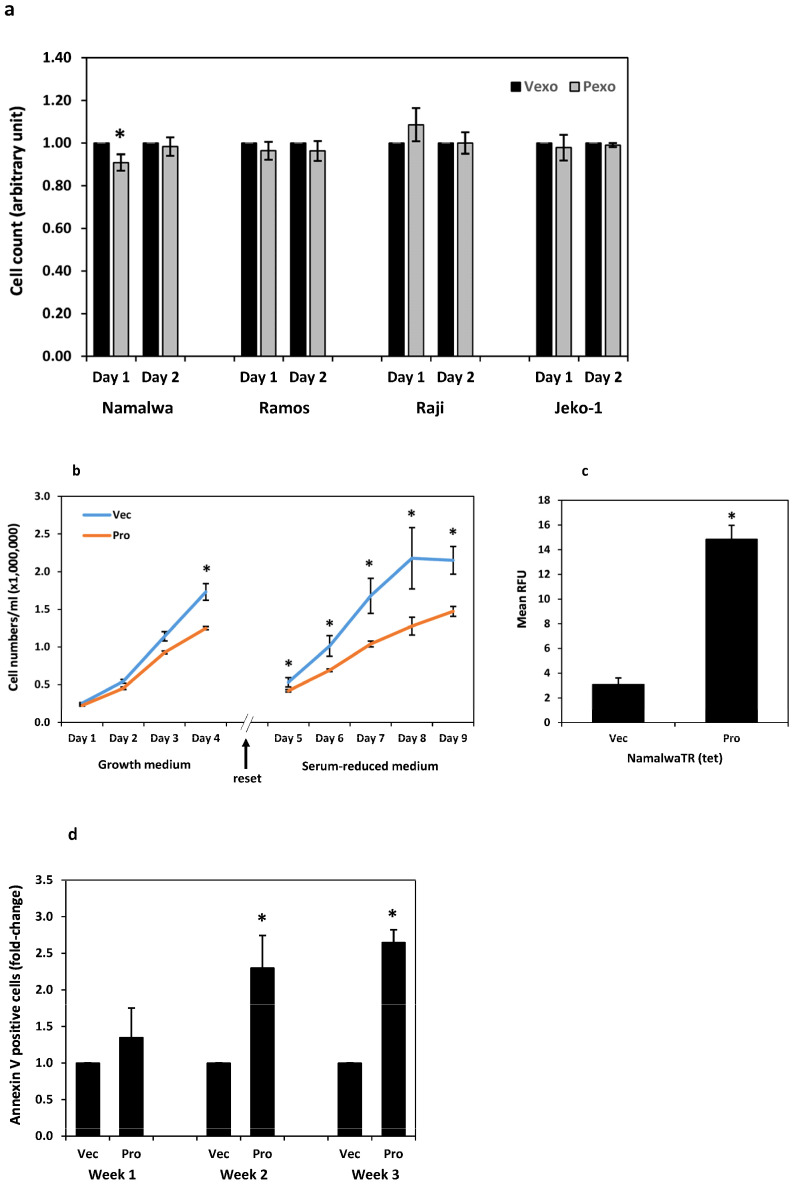

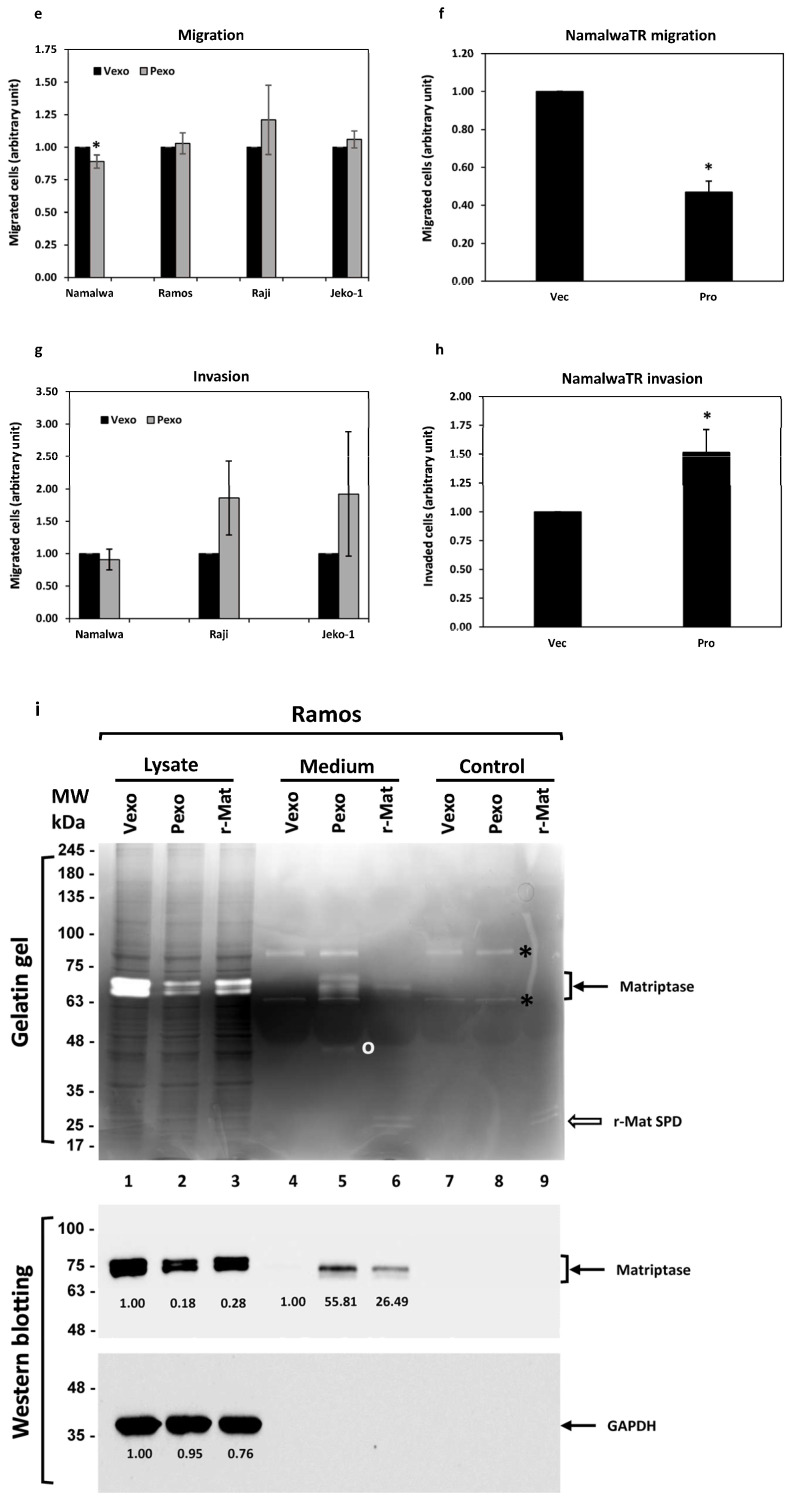

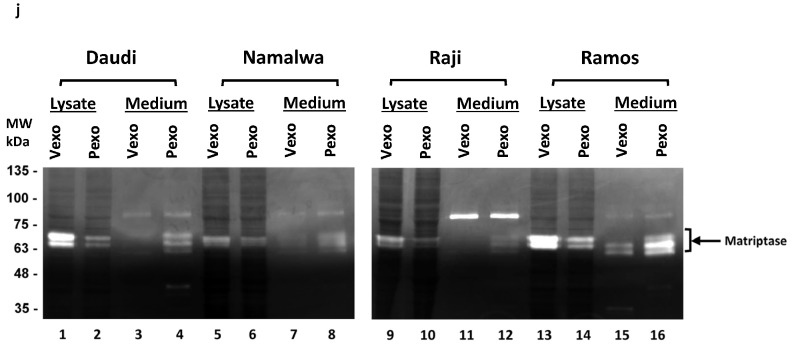
Impact of prostasin–matriptase cascade activation on B cancer cells. (**a**) Bar graph of cell count for two consecutive days of B cells treated with exosomes. Namalwa, *n* = 7; Ramos, *n* = 6; Raji, *n* = 5; Jeko-1, *n* = 6. * denotes *p* < 0.05. (**b**) Growth curves of NamalwaTR-Vec and NamalwaTR-Pro cells under tetracycline induction. Left graph, cells were set at 2.5 × 10^5^/mL on day 0 and cultured in the growth medium containing 10%FBS for 4 days. Right graph, on day 4 (reset, indicated by the arrow), the cells were diluted in OPTI-MEM I/2%FBS to 5 × 10^5^/mL and cultured for another 5 days. Tetracycline at 1 μg/mL was added into the culture on day 0 and maintained through culturing. *n* = 4 for each cell line, and * denotes *p* < 0.05. (**c**) Trypsin-like serine protease activity in the conditioned media of NamalwaTR-Vec and NamalwaTR-Pro cells (*n* = 4). Data were analyzed in Excel with student’s *t* test. * denotes *p* < 0.05 between the two sample groups. (**d**) Bar graph of annexin-V-positive cells analyzed by flow cytometry. Cells under tetracycline induction were cultured for various times (week 1, *n* = 3; week 2, *n* = 4; week 3, *n* = 3) and subjected to direct labeling of annexin V conjugated with fluorophore allophycocyanin (APC). Ten thousand cells for each sample were analyzed on the CytoFLEX S flow cytometer. Propidium iodide staining and FSC/SSC discrimination were used for gating the live singlets, which were further analyzed for annexin V staining. (**e**) Bar graph of migrated cells treated with exosomes for 24 h. * denotes *p* < 0.05. (**f**) Bar graph of migrated Namalwa sublines with the induction of prostasin expression for 2–4 days and reconditioned in RPMI medium for 1 day before seeding in Transwells for migration (*n* = 7). * denotes *p* < 0.05. (**g**) Bar graph of invaded cells treated with exosomes for 24 h. (**h**) Bar graph of invaded Namalwa sublines (*n* = 5) treated as in (**f**). * denotes *p* < 0.05. (**i**) Gelatin zymography and western blot analysis. Top panel, the Ramos cells (2 × 10^6^) in 500 μL of RPMI/0.1%BSA were treated with vector exosomes (Vexo), prostasin exosomes (Pexo), or a purified recombinant human matriptase serine protease domain (r-Mat SPD) overnight. One-fifth of the cell lysate (lanes 1–3) or 20 μL of the conditioned medium (lanes 4–6) were analyzed. The Vexo or Pexo exosomes or the r-Mat SPD alone were incubated in RPMI/0.1%BSA and used as controls (lanes 7–9). The clear bands at ~70 kDa marked by a filled arrow are matriptase. These were recognized by matriptase antibodies (middle panel). Unidentified bands with gelatinase activity marked at * locations in lanes 4, 5, 7, 8 are inherited from the exosomes, as shown in the samples with the exosomes alone (lanes 7 and 8). The band marked by the white circle is unknown. Bands at ~28 kDa marked by an unfilled arrow are r-Mat SPD. Bottom panel is GAPDH, which is detected only in the cell lysate, not in media samples or the controls without cells. (**j**) Gelatin zymography of B cancer cells treated as described in (**i**). The matriptase gelatinase activity is decreased in the cell lysate (lanes 2, 6, 10, 14) but increased in the corresponding media samples (lanes 4, 8, 12, 16) upon Pexo treatment in comparison to that of the Vexo-treated samples (in lysate, lanes 1, 5, 9, 13; in media, lanes 3, 7, 11, 15), correspondingly.

The Namalwa, Ramos, Raji, and Jeko-1 cells were cultured with prostasin exosomes (Pexo) or the control vector exosomes (Vexo) in a serum-reduced medium at a ratio of 1 unit of exosomes per 1 × 10^6^ cells in 500 µL. The number of cells in each sample was monitored by flow cytometry. Cell numbers were not significantly different in two days of the co-culture between the samples treated with prostasin exosomes and vector exosomes, except for the Namalwa cells. There was a slight decrease (by 9%) in cell number in the samples treated with prostasin exosomes in 24 h, in comparison to that treated with the vector exosomes (Figure 6a). When the NamalwaTR-Pro cells at a concentration of 2.5 × 10^5^ cells per milliliter were induced to over-express prostasin (Pro) with tetracycline (1 µg/mL), the growth rate of these cells decreased significantly on Day 4, with lower cell numbers in comparison to the tetracycline-treated NamalwaTR-Vec control cells (Vec) (Figure 6b, left graph). The cells were then diluted to a density of 5 × 10^5^ cells per milliliter and re-cultured for another five days in the serum-reduced medium. The growth rate of the NamalwaTR-Pro cells continued to decline, in comparison to the NamalwaTR-Vec cells in the same culturing condition (Figure 6b, right graph).

The prolonged presence of prostasin in B cancer cells reduced cell growth, a phenotype possibly associated with a sustained activation of the prostasin-matriptase cascade. Indeed, the conditioned media of the cells over-expressing prostasin (Pro) had higher trypsin-like serine protease activity than that of the cells with the empty vector (Vec) (Figure 6c). This result is also consistent with the data presented in Figure 4, in which the conditioned media of the cells treated with prostasin exosomes have higher trypsin-like serine protease activity than that of the cells treated with the vector exosomes. 

The active matriptase is toxic to cells [10,23,48,49,50] when its inhibitors, i.e., the HAIs are absent in the cells. Such is the case in most hematological cancer cell lines [31]. The annexin V protein is an established cellular marker for apoptotic cells with its high binding affinity to the anionic phospholipid phosphatidylserine (PS), which is localized to the outer leaflet of the plasma membrane in apoptotic cells [51,52]. We show in Figure 6d that the amount of cell-surface annexin V in the NamalwaTR-Pro cells increased during longer culturing times, in comparison to that in the NamalwaTR-Vec cells. The increased apoptosis of the NamalwaTR-Pro cells might have contributed at least partially to the reduced growth of these cells (Figure 6b). 

#### 3.6.2. Migration and Invasion

Physiologically, B cells migrate from one tissue to another, with chemotaxis following an increasing chemokine concentration gradient. We assessed the chemotactic migration ability of B cancer cells during the activation of the prostasin-matriptase cascade. Upon treatment with either prostasin exosomes or vector exosomes, the Ramos, Raji, and Jeko-1 cells did not have a significant change in migration through the membrane with a 5 μm pore size (Figure 6e). The Namalwa cells in the same setting had a slightly reduced migration (by ~11%). The Daudi cells did not migrate or invade under the same experimental conditions, and the RS4 cells did not express matriptase. Both cells were excluded here. For the NamalwaTR-Pro cells induced to over-express prostasin (Pro), a significant reduction in migration (by over 50%) was observed (Figure 6f) in comparison to the NamalwaTR-Vec cells. 

When the Matrigel matrix was constructed in the Transwell to assess the invasion properties of the B cancer cells under the influence of prostasin exosomes, there were no significant changes in invasion (Figure 6g). The Ramos cells did not invade efficiently through the Matrigel matrix under the same conditions. The NamalwaTR-Pro cells over-expressing prostasin showed a significant increase in invasion (by over 50%) through the Matrigel matrix (Figure 6h). A sustained prostasin–matriptase cascade activation appeared to decrease migration but increase invasion for the Namalwa cells in the presence of chemokines.

#### 3.6.3. Gelatinase Activity

Matriptase was reported to have gelatinase activity [44,53], which offers a direct method to visually monitor the matriptase expression and function regulation phenotypes associated with prostasin endowment in B cancer cells. We evaluated the matriptase gelatinase activity in gelatin gels following electrophoresis. As shown in Figure 6i (top panel, lane 1), matriptase in the Ramos cells treated with vector exosomes (Vexo) cleaved the gelatin in the gel, producing clear doublet bands after staining with Coomassie blue dye. The bands correspond to the matriptase protein, recognized by the matriptase antibody (Figure 6i, middle panel, lane 1). Due to the different conditions used for the gelatin gel, i.e., non-reducing and without sample boiling, the matriptase protein appeared at the ~70 kDa position in the gelatin gel, as opposed to the ~80 kDa position in the SDS-APGE/western blot. The prostasin exosome (Pexo) treatment reduced the matriptase level in the Ramos cells (Figure 6i, middle panel, lane 2) and correspondingly reduced the associated gelatinase activity (Figure 6i, top panel, lane 2). A purified recombinant human matriptase serine protease domain (r-Mat SPD, R&D Systems), when added (0.2 nM) in the Ramos cell culture, also reduced the quantity of the endogenous matriptase (Figure 6i, middle panel, lane 3) and the gelatinase activity of the endogenously expressed matriptase (Figure 6i, top panel, lane 3), suggesting that the soluble active matriptase can further activate more cellular matriptase zymogen. 

Prostasin exosomes mediated a reduction in the matriptase level in B cancer cells but an increase in the amount of matriptase in the culturing medium, with correspondingly increased gelatinase activity (Figure 6i, top panel, lane 5). The shed matriptase was recognized by the matriptase antibody (Figure 6i, middle panel, lane 5). Cells treated with the vector exosomes did not show very much matriptase released into the medium (Figure 6i, top and middle panels, lane 4). Cells treated with the r-Mat SPD released the endogenously expressed matriptase into the medium as well (Figure 6i, top and middle panels, lane 6). There was no detectable matriptase protein or gelatinase activity in samples with exosomes alone (lanes 7 and 8). The gelatinase activity of the r-Mat SPD was detected in samples either alone or in the Ramos cell culture medium (Figure 6i, top panel, lanes 9 and 6). 

The gelatinase activity of matriptase was also examined in the Daudi, Namalwa, and Raji cells, as shown in Figure 6j. Consistent with the results from the Ramos cells, the gelatinase activity of matriptase was low in the cells but high in the media after the prostasin exosome treatment in comparison to that of the cells treated with vector exosomes. The RS4 cells do not express matriptase and do not have matriptase-associated gelatinase activity (Figure S2). These results suggest that prostasin exosomes mediated the shedding of matriptase from the cell surface into the extracellular medium, whereas the shed-off matriptase retained its gelatinase activity, which could be involved in extracellular matrix modification. 

## 4. Discussion

Therapies targeting B-cell-specific markers have been developed in recent years for treating B cell diseases such as B cell lymphoma [54]. Rituximab is a monoclonal antibody developed to target CD20 on the B cell surface to remove aberrant B cells. MEDI-551, another monoclonal antibody, was developed to target CD19 on B cells to induce cytotoxicity. The required dose of MEDI-551 is lower than that of rituximab, and MEDI-551 depletes different subsets of plasma cells due to differential expression patterns of CD20 and CD19 on B cells. The monoclonal antibody epratuzumab targets CD22 to trigger signaling pathways in B cells and interfere with B cell proliferation. Other therapeutic strategies targeting B cell survival factors and mediators involved in intercellular and intracellular B cell functions are also in progress.

The prominent ectopic over-expression of matriptase, an otherwise epithelial-specific extracellular membrane serine protease in B cancer cells, presents a logical cell-surface molecule for targeting. In epithelial cells in which prostasin and matriptase are co-expressed, prostasin over-expression downregulates the matriptase level, and an insufficient prostasin expression level results in an upregulation of the matriptase level [42,47]. Upon zymogen activation, matriptase is shed from the cells, with an accumulation of the shed-off active matriptase in the culturing medium [20]. In most cases, the activated matriptase is almost immediately complexed with its inhibitor HAI-1, preventing further unwarranted matriptase activation and subsequent cytotoxicity. However, in hematological cancer cell lines, only 20% express both HAI-1 and HAI-2 in the same cells. In a subgroup of Burkitt lymphoma, HAI-1 is not expressed, with HAI-2 expressed at a very low quantity. 

Such a molecular landscape would suggest that the over-expressed matriptase in the B cancer cells is in the inactive zymogen form. If matriptase is activated in these B cells, it could initiate an untamed matriptase auto-activation cascade with a subsequent cytotoxicity triggered by a stressed Gogi-endoplasmic reticulum apparatus [23]. Given the peculiarity of matriptase over-expression in B cancer cells in the absence of prostasin, and absence or scarcity of HAIs, we hypothesized that an endowment of prostasin expression could initiate the proteolytic activation cascade. We aimed to use prostasin exosomes to achieve this, with a long-term consideration of developing a clinically feasible approach for alternative treatment of B cell lymphoma, based on the burgeoning momentum of exosomes on this front.

Our attempt at directing a prostasin action onto the B cancer cell surface via the exosomes appeared to have been successful, with a very clear phenotype of matriptase quantity and functional changes. This was achieved with prostasin exosomes prepared from a cell type with a full complement of relevant proteins, Calu-3 human lung cancer cells, expressing endogenous prostasin, HAI-1, and HAI-2; or HEK293T cells, with null or minimal expression levels of all three. The cellular matriptase content was greatly reduced, with a corresponding increase in the shed-off soluble enzyme in the culture medium. Along with the molecular changes, tumor cell behaviors were also modified, with phenotypes consistent with the tumor suppressor role of prostasin, such as reduced growth and increased apoptosis. It is not surprising that not all cell lines manifested a uniform phenotypic response to prostasin, given the nature of cancer cells. 

Using the various control conditions and prostasin variants as described, the action is more robust with GPI-anchored membrane-bound serine protease-active prostasin. It remains technically challenging to ascertain an actual molecular transfer of the exosome prostasin cargo onto the recipient B cells. But a prostasin-specific effect was supported by the lentiviral-transduced prostasin expression in the B cells, recapitulating the exosome delivery phenotypes. The lentiviral-transduced prostasin expression also allowed us to tease out a long-term phenotype associated with prostasin expression in B cancer cells, i.e., growth inhibition (Figure 6b).

The introduction of prostasin to the B cancer cells clearly resulted in the release of matriptase into the culture medium (Figure 2a), which had a corresponding gain of trypsin-like serine protease activity, as measured by the cleavage of the synthetic substrate QAR-AMC (Figure 4a). The QAR (P3-P1) is a highly selective matriptase and prostasin substrate that has been routinely used to demonstrate proteolysis by these specific serine proteases in complex sample contexts, such as cell lysate or living cells [25]. The B lymphoma cell lines used in this study were all validated for QAR selectivity toward matriptase in the conditioned media upon acid activation by a previous study [31]. The conditioned media tested for QAR cleavage would not have very appreciable amounts of prostasin, which would be even across the different cell types if there were residual amounts from the exosome treatment. The QAR-cleaving activities in the conditioned media at different levels across the cell types may thus be attributed to the shed-off active matriptase. The cellular matriptase in the B cancer cells was considered to be in the zymogen form, but in the gelatin zymography experiment, it showed gelatinase activity in the cell lysate (Figure 6i). The gelatin-degrading matriptase in the gel in situ underwent SDS denaturation during electrophoresis and renaturation for zymography. This process may very well have resulted in the activation of the enzyme in situ. Such a phenomenon was documented for the zymogen of trypsin (trypsinogen), showing a strong activity in gelatin zymography performed under conditions similar to those used here [55].

A question that arises and also remains concerns the fate and function of the shed-off soluble active matriptase upon the introduction of prostasin in the B cancer cells. We were able to induce a similar response with the use of a soluble recombinant human matriptase protease domain (Figure 6i). Thus, we may speculate that the shed-off soluble active matriptase would be able to do the same. We may also expect that newly synthesized matriptase zymogen would be perpetually subjected to a cycle of proteolytic activation and shedding, independent of the initial prostasin trigger. The data presented in Figure 4c with a sustained high trypsin-like serine protease activity in the culture medium over 10 days past the exosome treatment would be supportive of this. 

We should be cautious when interpreting the results of the in vitro experiments. The active matriptase is a well-known tumor progression promoter by activating growth factors for cell migration. However, in our study, the advent of matriptase activation triggered by prostasin promotes invasion, but it also inhibits migration and induces apoptosis. The prostasin-mediated matriptase activation in B cancer cells may have different outcomes in an in vivo context. In solid tumors of B cell lymphoma, the active matriptase may be accumulated in situ, playing roles in the tumor microenvironment. The sustained matriptase activation could modify the extracellular matrix by degrading matrix proteins, such as collagen IV, or acting on its substrates, such as the urokinase plasminogen activator (uPA) and hepatocyte growth factor (HGF) in the neighboring cells, especially the fibroblasts [56,57]. On the other hand, the accumulation of active matriptase in the tumor microenvironment may also induce cell apoptosis, as the active matriptase is toxic to the cells [10,23,48,49,50]. 

In this study, prostasin exosomes could activate matriptase in all B cells tested here, as evidenced by the reduced cellular matriptase, increased trypsin-like serine protease activity in the conditioned media, and the accumulation of soluble matriptase therein. The matriptase in the JeKo-1 cells was reported as not being able to go through auto-activation induced by acid [31], but it could apparently be activated by the prostasin exosomes, as we have shown (Figure 4). This may suggest that different activation mechanisms were involved in the two experimental conditions. 

Our study focused on a limited selection of four Burkett lymphoma cell lines with high levels of matriptase protein expression in the absence of its physiological inhibitor HAI-1 [31], which is also an inhibitor of prostasin. In future studies, we may consider testing the prostasin exosome activation of matriptase in cell lines representing the most common subtype of NHL, i.e., diffuse large B-cell lymphoma (DLBCL), such as OCI-LY3 and OCI-LY10. These have different molecular genetic and gene expression backgrounds than the Burkett cell lines we investigated, especially with regard to the expression of HAI-1 [31]. This would be very informative as to the potential role of HAI-1 in the prostasin-exosome-induced matriptase activation in these cell lines, with an abundance of the HAI-1 protein in the OCI-LY10 cells but an absence in the OCI-LY3 cells.

## 5. Conclusions

Our study began with the question as to what would happen if we imposed prostasin expression in B cancer cells ectopically expressing the otherwise epithelial-specific matriptase. The hypothesis was that matriptase would be activated by prostasin, shed from the cells, and reduced in quantity, recapitulating the phenotypes previously reported with matriptase expression silencing using siRNA. Our results supported this hypothesis. Prostasin-exosome-mediated matriptase removal from B cancer cells is an attractive and novel idea, shown to be efficient in the in vitro setting in this study. The use of an exosome-mediated proteolytic cascade to kill cancer cells could be exploited as an alternative approach to treat B cell lymphoma, but this requires validation in vivo, e.g., in xenografted tumors using animal models.

## Data Availability

The data presented in this study are available in this article.

## Supplementary Materials

The following supporting information can be downloaded at: https://www.mdpi.com/article/10.3390/cancers15153848/s1, File S1: The original western blots; Figure S1: Western blot analysis of exosomes. The vector control exosomes (Vexo, 10 μg) and prostasin exosomes (Pexo, 10 μg) prepared from the HEK293T cells were tested with four different exosome markers using antibodies against CD63, HSP70, Tsg101, and Alix (all from Santa Cruz Biotechnology). Lanes 1 & 2, CD63; lanes 3 & 4, HSP70; lanes 5 & 6, Tsg101; lanes 7 & 8, Alix. The panel of HSP70 membrane was stripped and re-blotted with the prostasin antibody (lanes 9 & 10). In a different experiment (right panel, lanes 11–14), the exosomes were incubated with 0.5 μg of purified mouse protease nexin 1 (PN-1) for 2 h at 37 °C before SDS-PAGE/western blotting; Figure S2. Gelatin zymography and western blot analysis of B lymphoma cell lines. Lysate from 2 × 10^5^ cells of each line was analyzed in gelatin gel (left panel), western blotting with matriptase antibody (AF3946, middle panel) and western blotting with GAPDH antibody (right panel). Lanes 1, RS4; lanes 2, Daudi; lanes 3 Namalwa; lanes 4, Raji; lanes 5, Ramos; lanes 6, Jeko. RS4 cells do not express matriptase (middle panel, lane 1) and do not have matriptase-associated gelatinase activity (left panel, lane 1).
