# Supplementary material for: Exosome-Mediated Activation of the Prostasin-Matriptase Serine Protease Cascade in B Lymphoma Cells
Source: Cancers (Basel). 2023 Jul 28;15(15):3848. doi: 10.3390/cancers15153848 (PMC10417574; doi:10.3390/cancers15153848)
Supplement: Supplementary file 1 [file cancers-15-03848-s001.zip › cancers-2496948-File S1.pdf]

All uncropped images are overlays of antibody blot & protein staining with MW markers)

Figure 1c Uncropped

Matriptase

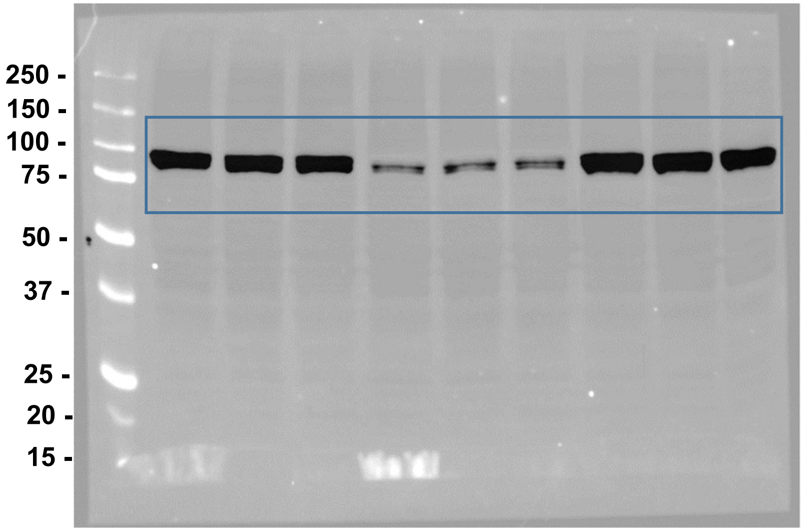

HAI-2

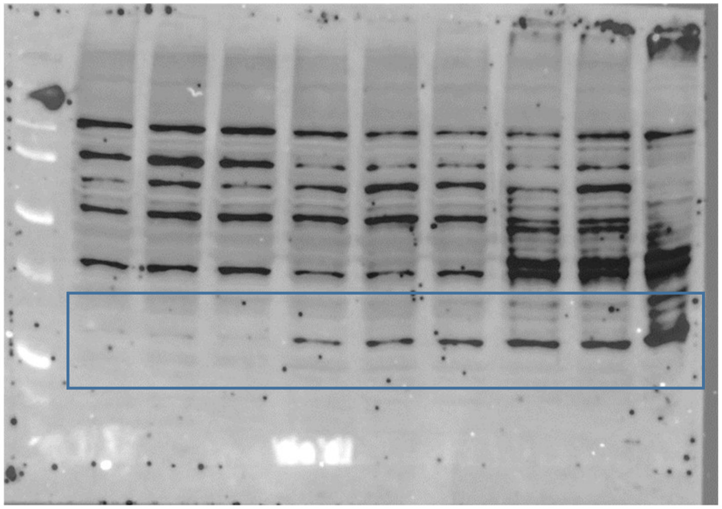

GAPDH

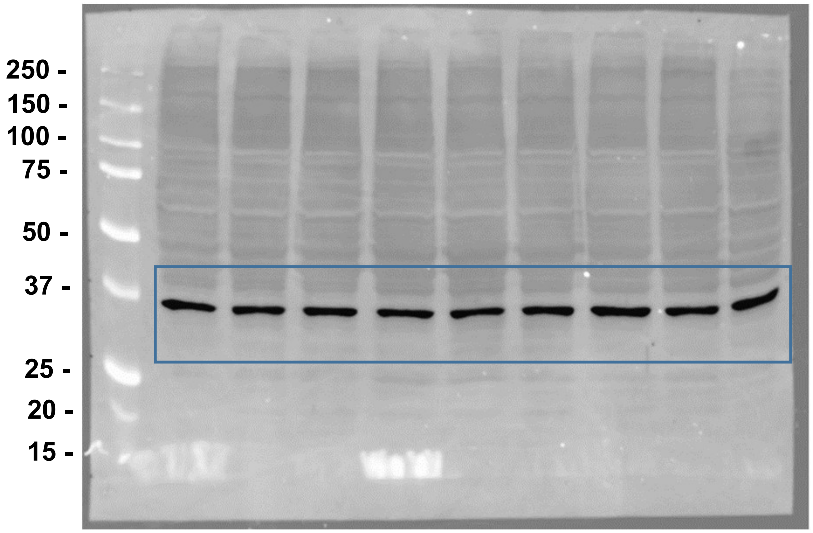

Figure 2a,b Uncropped

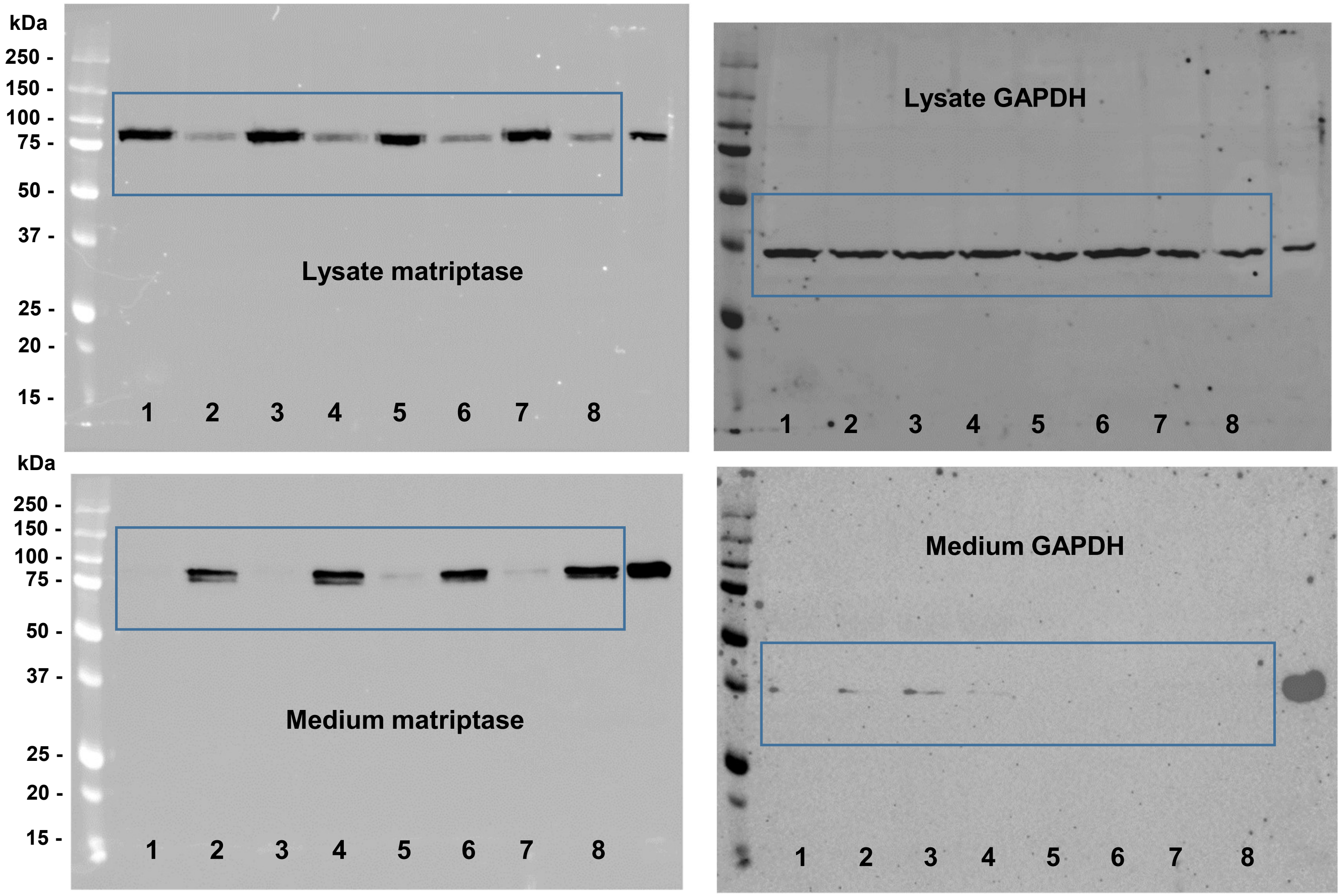

Figure 2e,g Uncropped

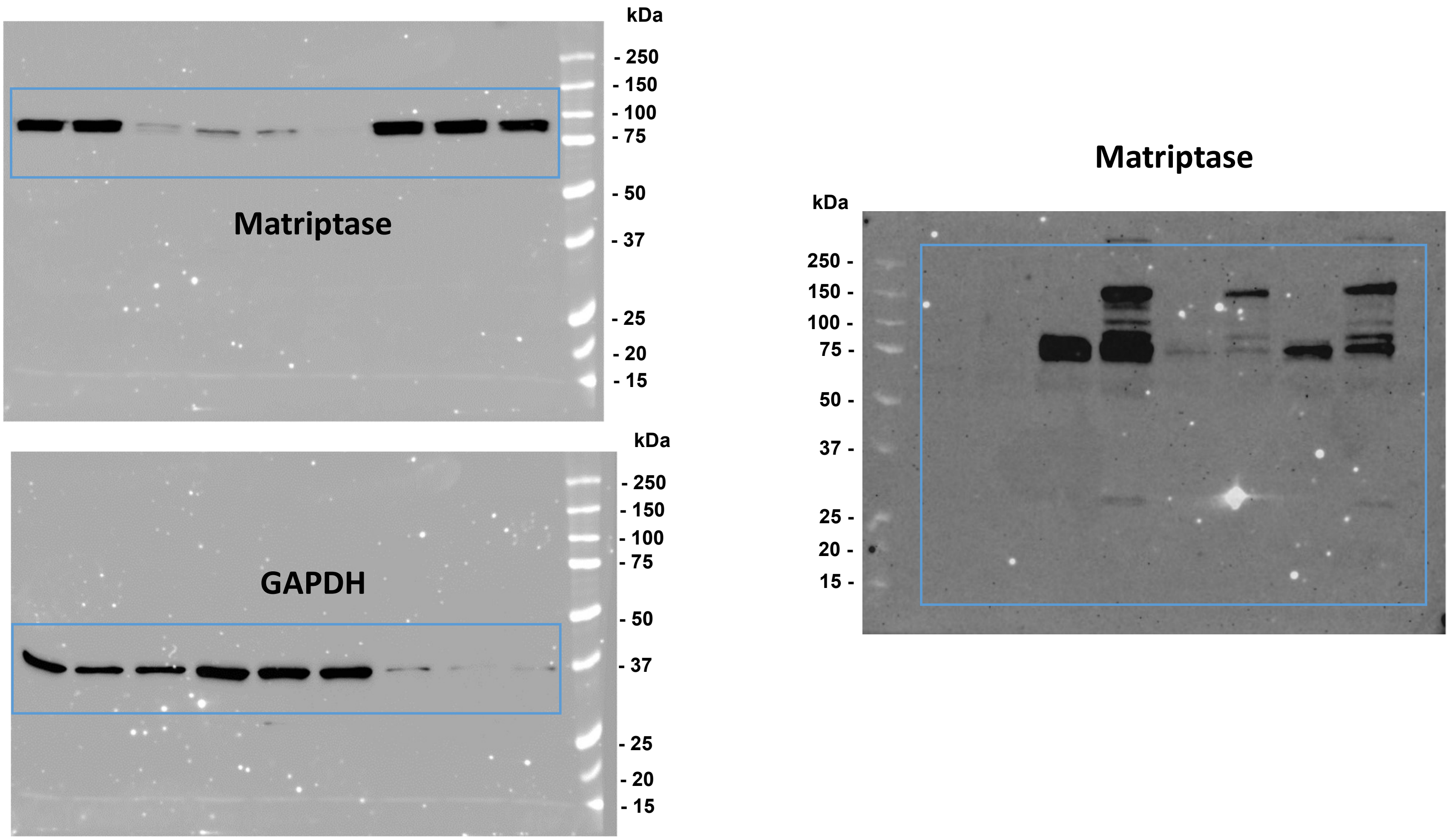

Figure 3a Uncropped

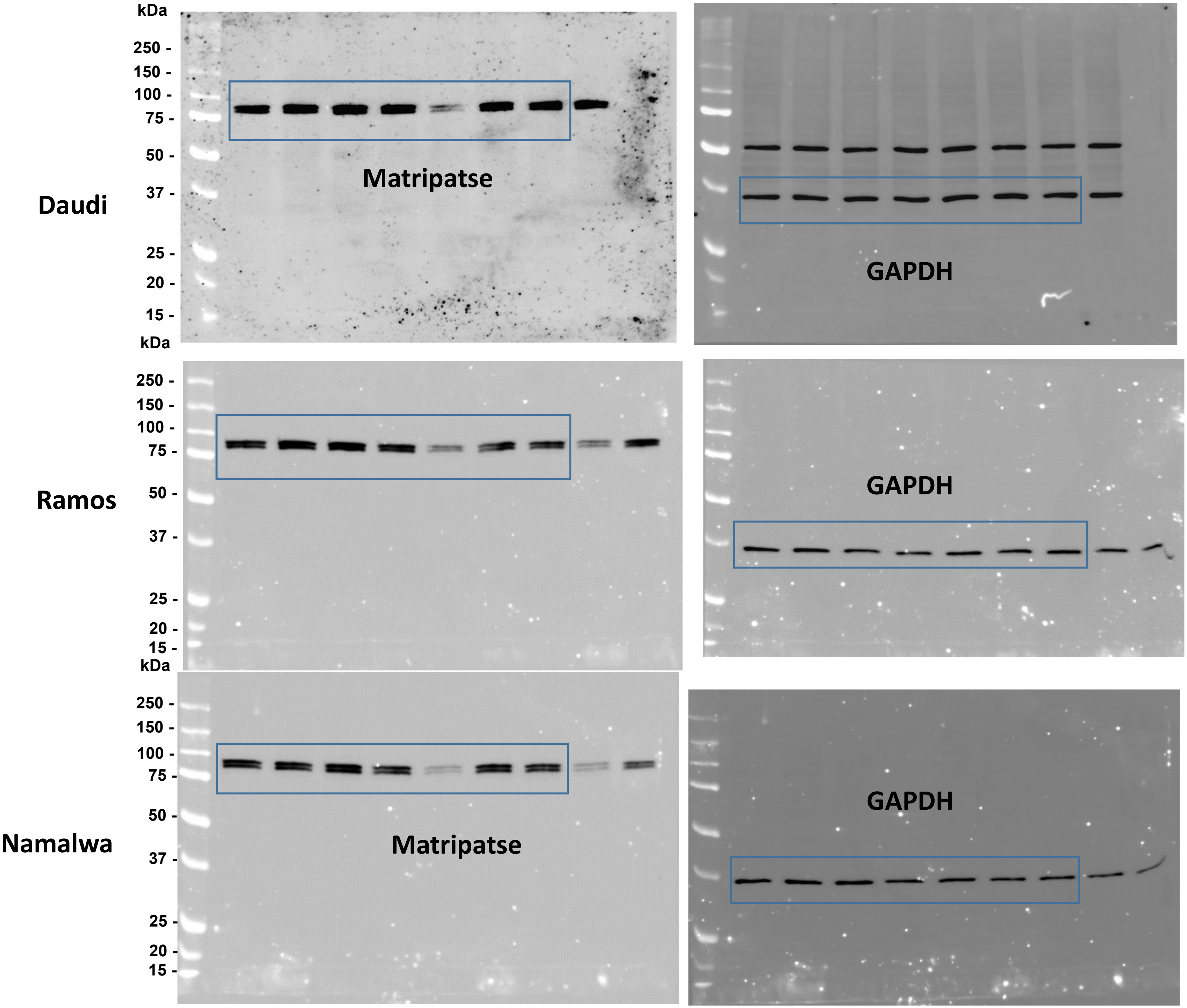

Figure 5a,c,d Uncropped

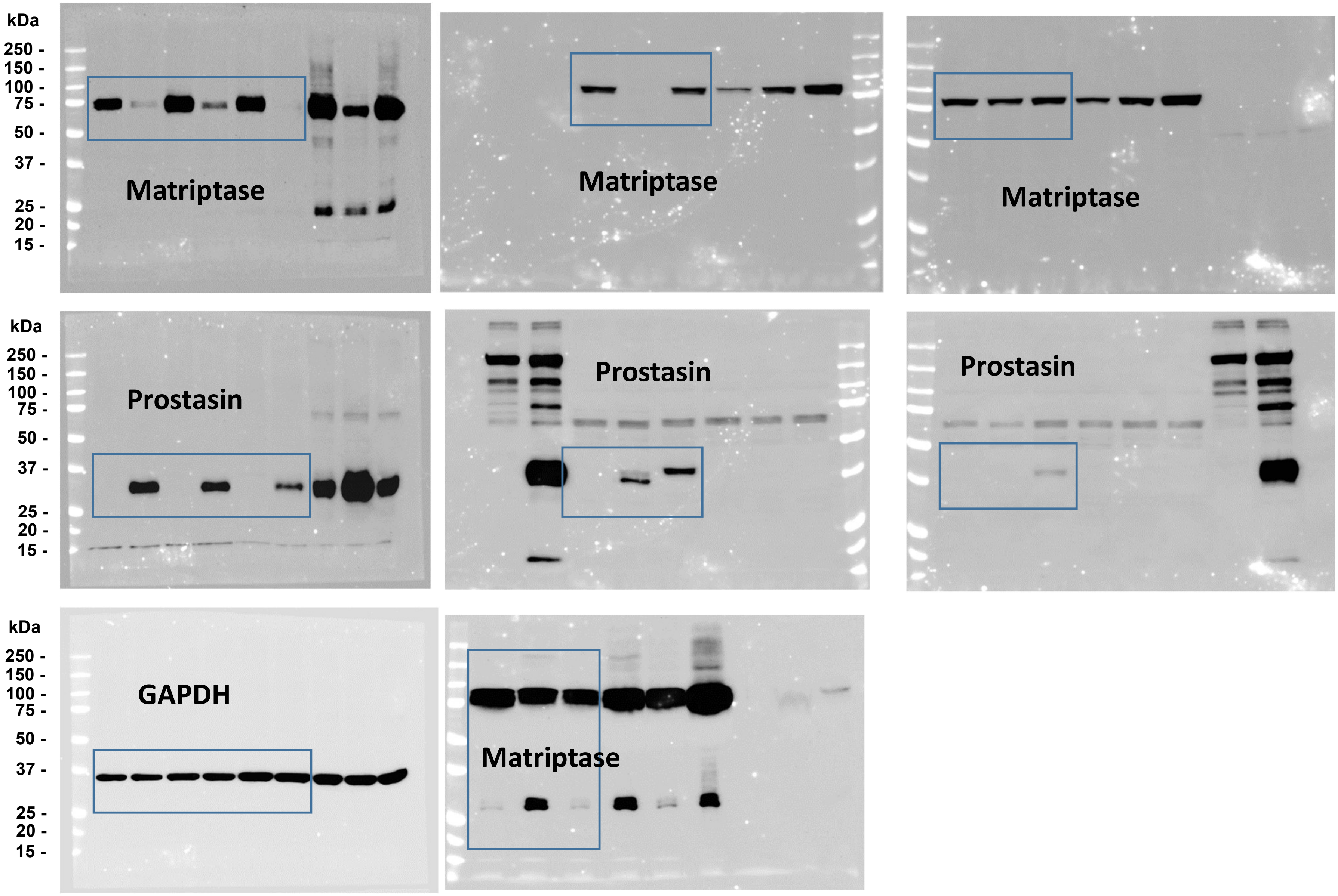

Figure 6i Uncropped

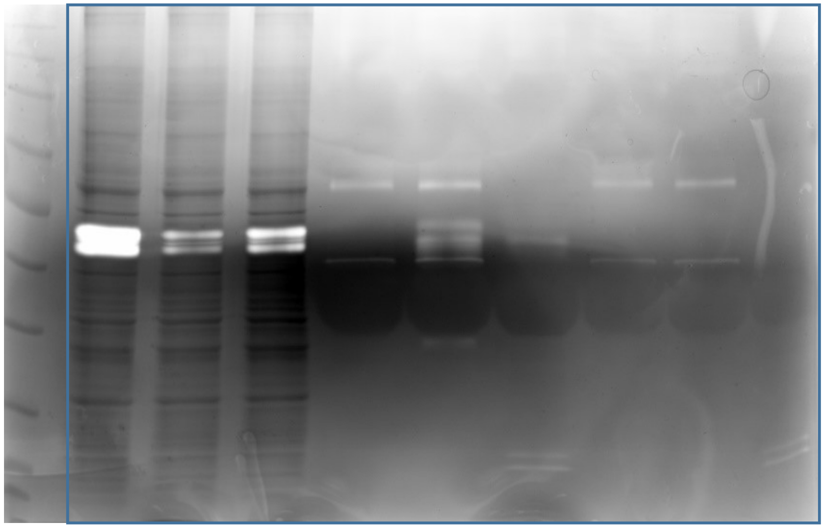

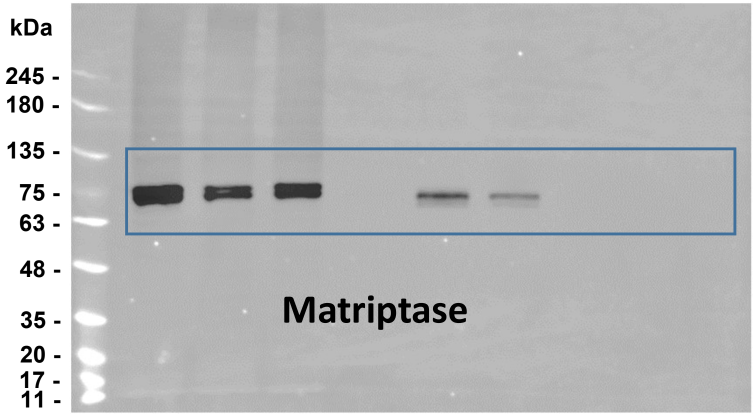

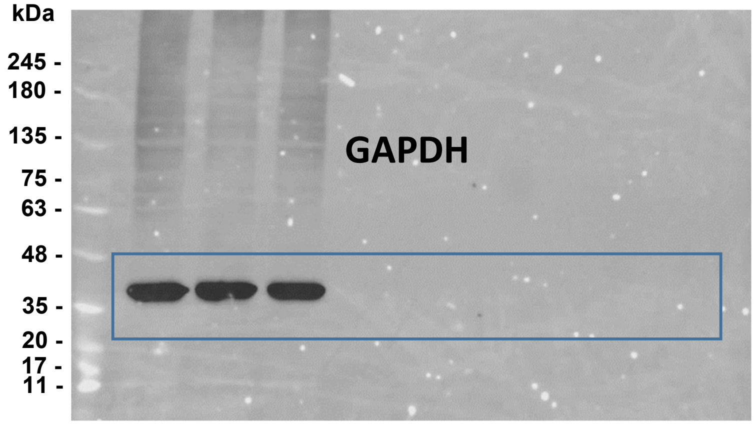

Figure 6j Uncropped

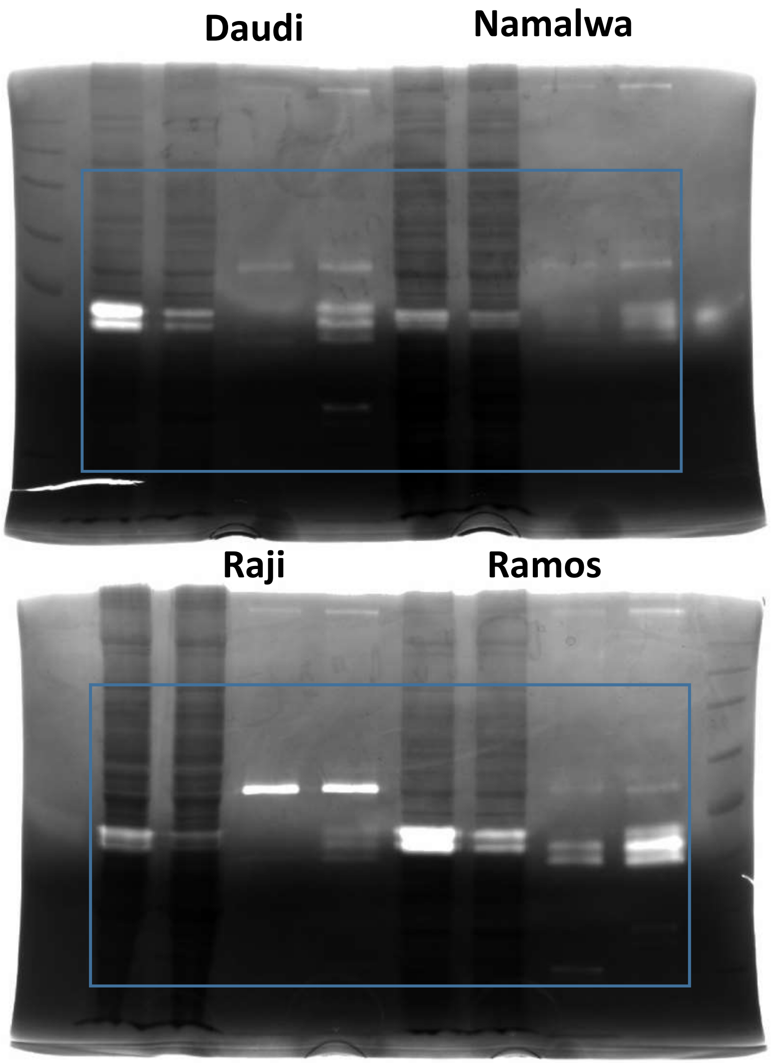

Figure S1 Uncropped

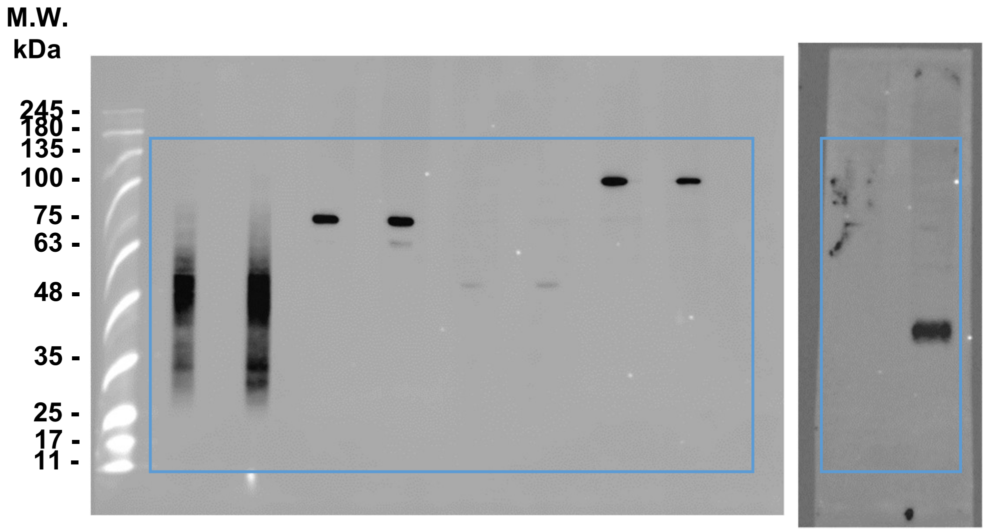

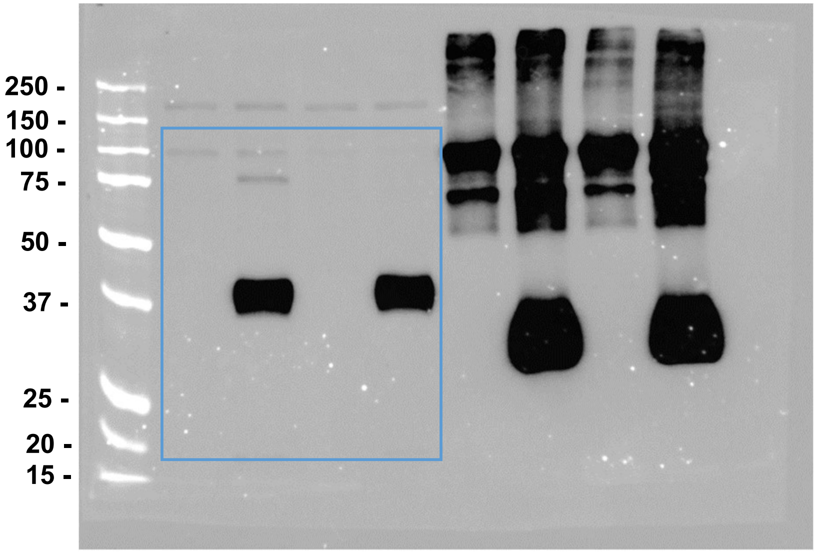

Figure S2 Uncropped

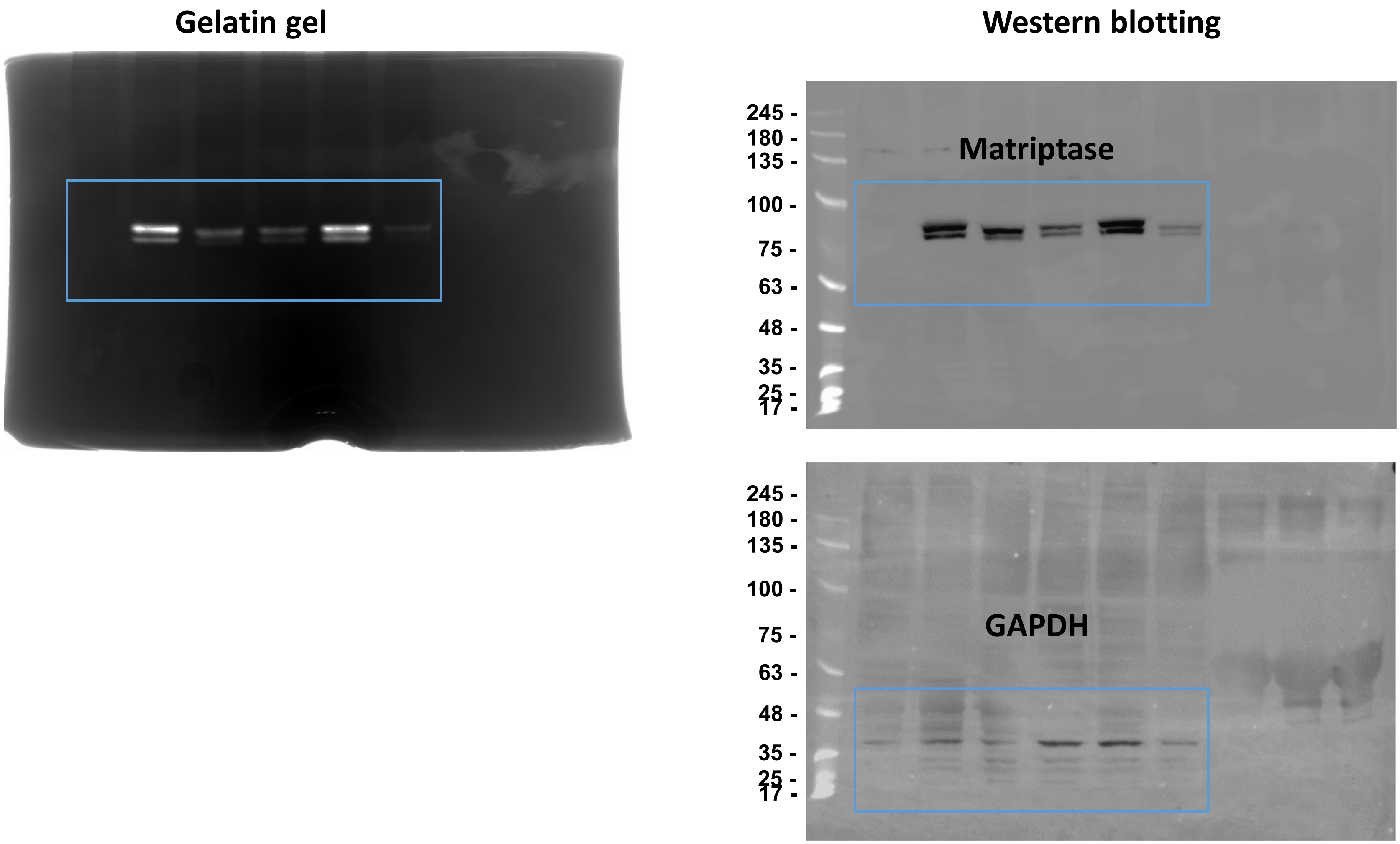

File S1. The original western blots.
