# Supplementary material for: Exosome-Mediated Activation of the Prostasin-Matriptase Serine Protease Cascade in B Lymphoma Cells
Source: Cancers (Basel). 2023 Jul 28;15(15):3848. doi: 10.3390/cancers15153848 (PMC10417574; doi:10.3390/cancers15153848)
Supplement: Supplementary file 1 [file cancers-15-03848-s001.zip › cancers-2496948-supplemntary figures.pdf]

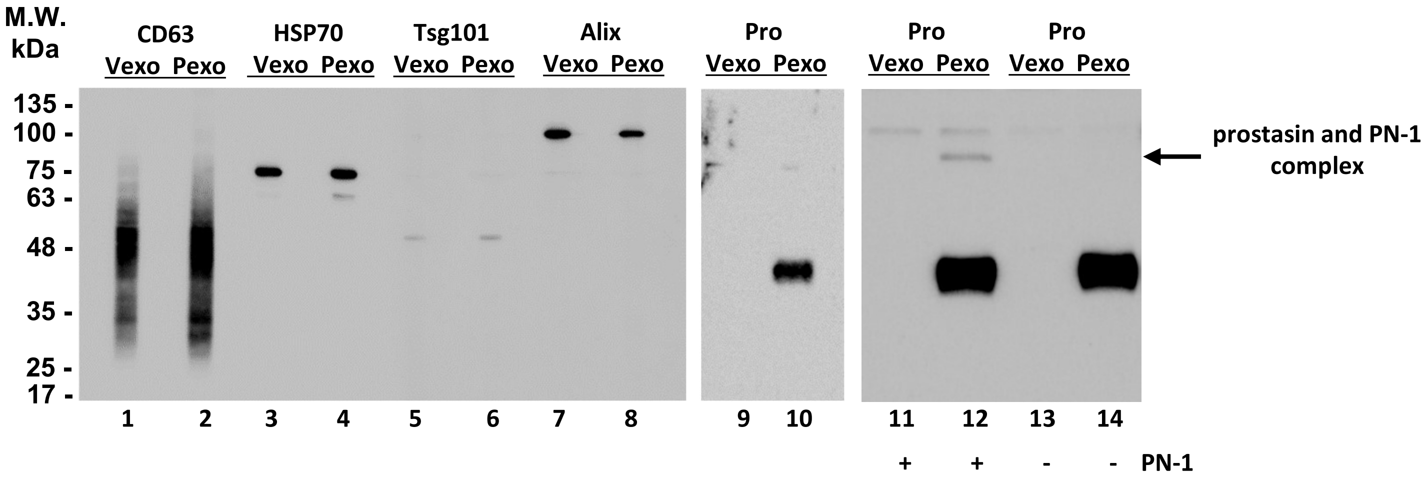

Figure S1. Western blot analysis of exosomes. The vector control exosomes (Vexo, 10 μg) and prostasin exosomes (Pexo, 10 μg) prepared from the HEK293T cells were tested with four different exosome markers using antibodies against CD63, HSP70, Tsg101, and Alix (all from Santa Cruz Biotechnology). Lanes 1&2, CD63; lanes 3&4, HSP70; lanes 5&6, Tsg101; lanes 7&8, Alix. The panel of HSP70 membrane was stripped and re-blotted with the prostasin antibody (lanes 9&10). In a different experiment (right panel, lanes 11-14), the exosomes were incubated with 0.5 μg of purified mouse protease nexin 1 (PN-1) for 2 hours at 37°C before SDS-PAGE/western blotting.

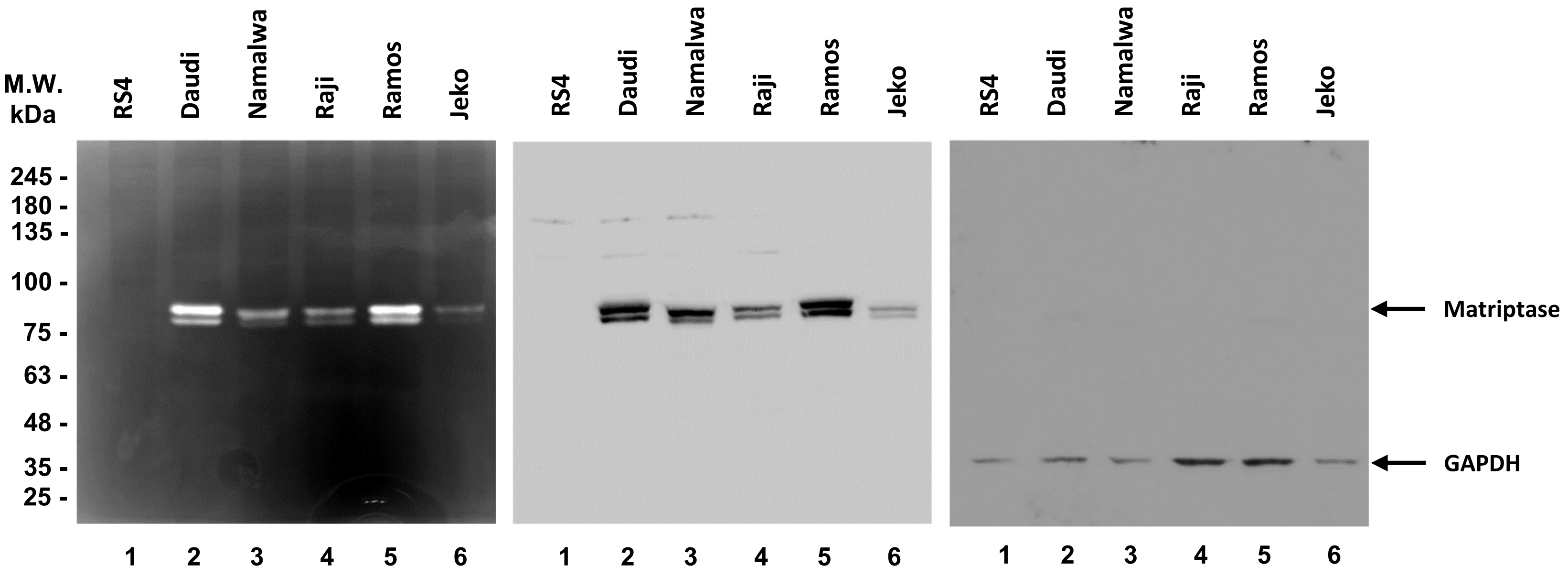

Figure S2. Gelatin zymography and western blot analysis of B lymphoma cell lines. Lysate from $2x10^5$ cells of each line was analyzed in gelatin gel (left panel), western blotting with matriptase antibody (AF3946, middle panel) and western blotting with GAPDH antibody (right panel). Lanes 1, RS4; lanes 2, Daudi; lanes 3 Namalwa; lanes 4, Raji; lanes 5, Ramos; lanes 6, Jeko. RS4 cells do not express matriptase (middle panel, lane 1) and do not have matriptase-associated gelatinase activity (left panel, lane 1).
